# Microbial interactions mediate the fairy ring type effects on alpine meadow plant communities on the Tibetan plateau

**DOI:** 10.1186/s40793-026-00873-z

**Published:** 2026-03-10

**Authors:** Zhihua Wu, Shiting Cao, Sisong Tu, Yuying Zhang, Ming Wang, Huiyuan Ren, Chengxing Wu, Shilong Chen, Qingbo Gao, Rui Xing

**Affiliations:** 1 Northwest Institute of Plateau Biology, Chinese Academy of Sciences, 23# Xinning Lu, Xining, 810008 Qinghai China; 2 College of Life Science, University of Chinese Academy of Sciences, Beijing, 100039 China; 3 Qinghai Nationalities University, 3# Bayizhonglu, Xining, 810007 Qinghai China; 4 Qinghai Provincial Key Laboratory of Crop Molecular Breeding, 23# Xinning Lu, Xining, 810008 Qinghai China

**Keywords:** Fairy ring, Tibetan plateau, Alpine meadow, Microbial interactions, Mushrooms

## Abstract

The Fairy ring (FR) is a circular formation created by the mycelium of the FR fungus during its dynamic proliferation in the soil. FRs were categorized into three types based on their varying effects on adjacent plants. Type I FRs (T1) cause extensive damage with wilting and necrosis, Type II FRs (T2) promote growth with dark-green zones, and Type III FRs (T3) feature fruiting bodies with minimal impact on vegetation. T2 FR often display conspicuous growth-promoting zones and thus are studied more frequently than other types. Here we compare three FR types—*Agrocybe* sp. (T1), *Agaricus campestris* (T2), and *Clitocybe* sp. (T3)—across zones (ON (beneath the fruiting body)/IN (20 cm inward from the ON zone) /OUT (20 cm outward from the ON zone) and depths (0–10/10–20 cm) in Tibetan alpine meadows using 16 S and ITS amplicon sequencing. We infer putative microbe–fungus associations from diversity patterns, differential abundances, and co-occurrence analyses. Our research revealed that bacterial diversity was minimal in the ON zones; in contrast, fungal diversity was maximal, which may be attributable to the selection and interspecific competition among FR fungi. Taxa enriched in ON zones differed among FR types: T1 exhibited enrichment of Nitrospiria (nitrification) and Phycisphaerae (organic matter decomposition); T2 demonstrated enrichment of Actinobacteria and Proteobacteria, correlating with plant-growth enhancement; T3 displayed diminished recruitment, consistent with negligible vegetation influence. Cross-kingdom co-occurrence networks demonstrated increased microbial collaboration in ON zones, especially in surface soils for T1/T3 and in subsurface layers for T2. Different types of FR fungi exert varying effects on soil microorganisms at different depths. This study provides comparative data on three types of fairy rings and their associated soil microorganisms, offering insights into elucidating the potential mechanisms of fungal-bacterial-plant interactions. However, further validation through metabolomics and controlled experiments is still required.

**Supplementary Information:**

The online version contains supplementary material available at 10.1186/s40793-026-00873-z.

## Background

The Fairy ring (FR) is a natural phenomenon in which fungi develop in a circular formation, resulting in rings or arcs within meadows, woodlands, and artificial grassland environments [1]. Over 50 genera of fungi are capable of producing FRs, including *Agaricus*,* Agrocybe*,* Amanita*,* Boletus*,* Bovista*,* Calvatia*,* Cantherellus*,* Chlorophyllum*,* Clitocybe*,* Collybia*,* Lactarius*,* Lepiota Leucocalocybe*,* Lycoperdon*,* Clitocybe*,* Collybia*,* Floccularia*,* Hebeloma*,* Hydnum*,* Hygrophorus*,* Lactarius*,* Lepiota*, and *Vascellum*, etc. [2, 3, 4]. The typical FR, as it is generally recognized, can be visible as circles of lush, dark green grass; in the early twentieth century, Shantz and Piemeisel (1917) categorized FRs into three categories based on their varying impacts on plant development [3]. Type I FRs (T1) cause the most extensive damage, characterized by wilting, necrotic, or deceased grass [5]. Type II FR (T2) forms dark-green, growth-promoting zones [6]. Type III FR (T3) features fruiting bodies with minimal effects on vegetation [7]. Despite extensive work on T2, T1, and T3 remain comparatively understudied, particularly regarding belowground microbiomes across ring zones and soil depths.

Subsequent research has focused on the impact of the FR fungus on the physicochemical properties of adjacent soils, elucidating the mechanisms by which it affects plant growth conditions. Fisher [8] discovered that the FR fungus *Marasmius oreades* facilitates the mobilization of nitrogen and phosphorus during mycelial growth, thereby influencing soil nutrient availability and potentially affecting plant health [8]. (Edwards [9], [10]) showed nutrient hotspots within *Agaricus arvensi*s rings [9, 10]. In the temperate steppe, *Agaricus gennadii* reshapes soil–plant stoichiometry [11]. Beyond nutrient mobilization, FR fungi produce bioactive metabolites, including imidazole-4-carboxamide (ICA) and 2-azahypoxanthine (AHX) that modulate plant development [12, 13].

Soil microorganisms are crucial for sustaining the health and functionality of soil ecosystems [14], their activity in the soil enhances soil structure and fertility [15]. Microbes decompose organic matter, cycling nutrients back into the soil, which is crucial for both natural ecosystems and agricultural productivity [16]. More recently, attention has shifted from soil chemistry to the microbial dimension of FR systems, exploring how fungal activity reshapes soil bacterial and fungal communities. With the advent and falling cost of high-throughput sequencing, studies increasingly profile microbial communities across FR zones—most commonly in T2. Collectively, these results indicate that the FR fungi influence the diversity and structure of adjacent soil microbial communities. The extent of this impact differs among FRs created by various fungus species, with inconsistencies also observed in findings from separate studies of the same species. Most studies have found that FR fungi can significantly impact the community structure of soil microorganisms within the growth-enhancing zone. These fungal species include *Agaricus gennadii* [17], *Calocybe gambosa* [18], *Tricholoma mongolicum* [19] and *T. matsutake* [20]. Together, these studies indicate changes in the soil physicochemical properties and metabolites associated with FRs, leading to significant alterations in the bacterial community. however, exceptions exist: some *T. matsutake* rings show little compositional change or ɑ-diversity differences [21].

The Tibetan Plateau, often referred to as the “Third Pole” because of its high elevation and extreme climate [22], hosts vast alpine meadows that play critical roles in biodiversity and ecosystem stability [23]. The conservation and sustainable management of these meadows are essential for both local and regional sustainability, as well as for global environmental health [24]. Research on FRs in Tibetan alpine meadows has emerged only in recent years. Wang et al., (2006) initially investigated the impact of the FR of *Floccularia luteovirens* on plant variety and soil physicochemical parameters on the Tibetan plateau [25]. In 2018, we initially examined the influence of FRs of *F. luteovirens* on soil microbial community structure by high-throughput sequencing and discovered a considerable reduction in microbial diversity beneath the FRs. Substantial alterations in soil physicochemical characteristics precipitated this transformation [26]. The research by Cao et al. [27] indicated that the influence of *F. luteovirens* on plants and soil microbes is facilitated by modifications in glucose and amino acid metabolism [27]. Du et al. [28] investigated the production of FRs by *Agaricus xanthodermus* in alpine meadows on the Tibetan plateau, noting that the impact of FRs on plants and microorganisms is contingent upon the habitat type [28]. Despite this progress, studies have primarily targeted Type II FRs, with limited attention to Type I and III [29]. As a result, the comparative effects of different FR types on soil microbial communities and their underlying mechanisms remain poorly understood. The study of microbial ecology should encompass not only alterations in community composition but also the interactions among microorganisms within a complex community [30]. The dynamic expansion of fungi in FRs offers an exemplary subject for investigating microbial interactions during this process. Here, we compared three representative FR types: *Agrocybe* sp. (T1), *Agaricus campestris* (T2), and *Clitocybe* sp. (T3) across spatial zones (beneath the FRs (ON), within (IN), and outside (OUT) the FRs) and soil depths (0–10, 10–20 cm), to address two main questions: (1) Do different FR fungi exert similar or divergent effects on soil microbial communities? (2) How do microorganisms interact with each other during the dynamic process of ring expansion?

## Methods

### Description of sampling sites

**Fig. 1:**
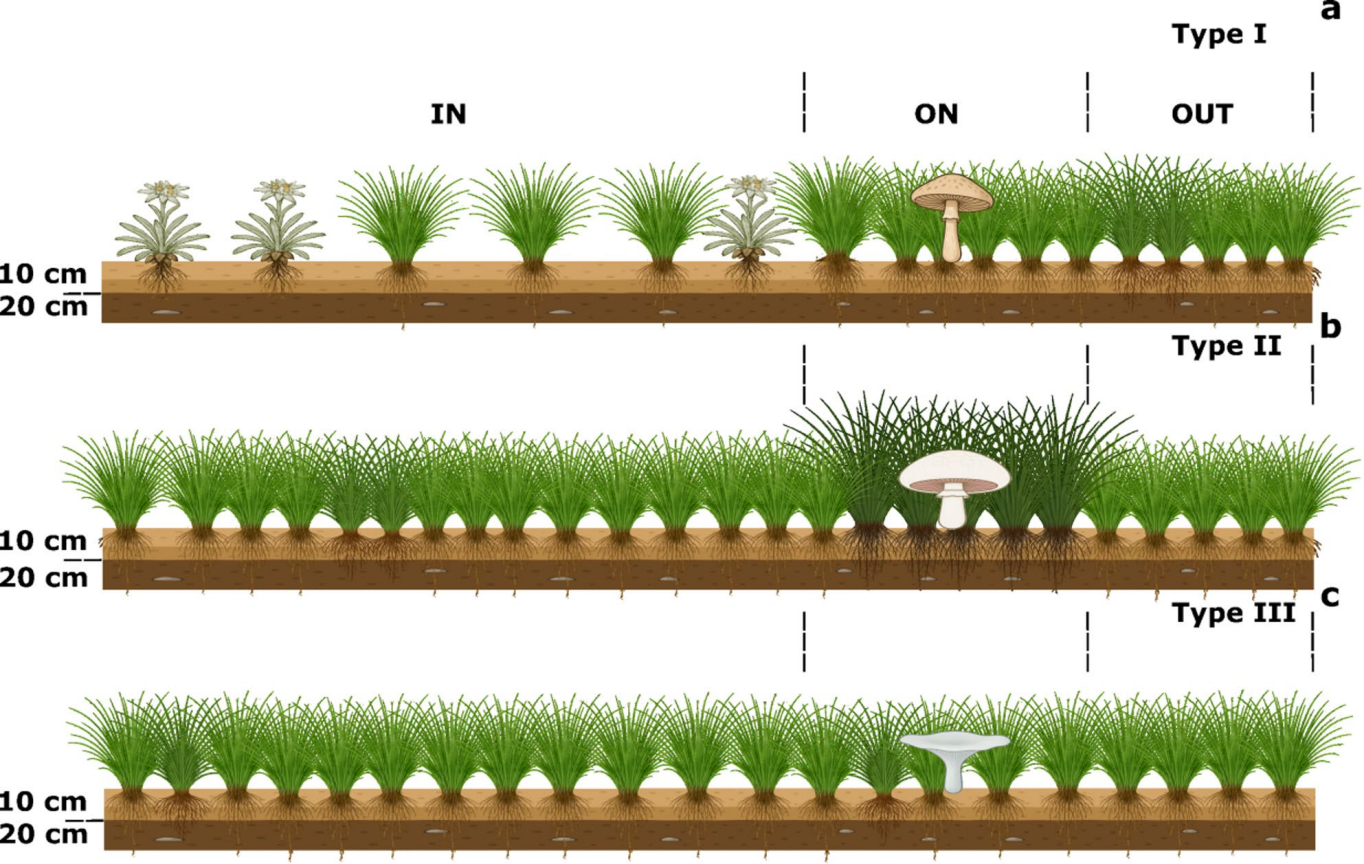
Sampling diagrams of three types of FRs. Type I FRs (with a significant decrease in plant coverage within the FR) are formed by *Agrocybe* sp. (**a**), Type II FRs (with visually visible promotional bands) are formed by *A. campestris* (**b**), and Type III FRs (where only a ring structure consisting of the fungal fruiting bodies can be observed) are formed by *Clitocybe sp.* (**c**). We collected soil samples (0–10, 10–20 cm) from three zones of the FR, where the ON zone is the zone under the substrate, the IN zone is the zone 20 cm inward from the ON zone, and the OUT zone is the zone 20 cm outward from the ON zone, and we also collected soil at different depths (0–10 com, 10–20 cm)

The sampling sites were located in alpine meadows on the northern edge of the Tibetan plateau (Yeniugou, Qinghai Province), and the site descriptions are identical to those in our investigation of the FR of *F. luteovirens* in the same area [26]. Fruiting body collection took place between July and September 2020, in accordance with the varying emergence seasons of different species. Specifically, samples of *Acrocybe* sp. were collected in late July 2020, *A. campestris* samples were collected in mid-August 2020, and *Clitocybe* sp. samples were collected in early September 2020. To reduce potential microbial fluctuations, efforts were made to collect samples of the same FR within a single day whenever possible (Table S1). The three FRs were T1 (Fig. 1a) formed by *Agrocybe sp.* (The dominant species on and outside the FRs are *Poa annua*, *Thermopsis lanceolata*, and *Stipa purpurea*, but inside the FRs, the only dominant species was *Leontopodium nanum* due to the effect of FR fungus). T2 (Fig. 1b) formed by *A. campestris* (a significantly stimulated belt on the FRs can be observed). T3 FRs (Fig. 1c), produced by *Clitocybe* sp., exhibited no substantial variations in plant composition across different zones of the FR; only the ring structure created by fungal fruiting bodies was evident. Details regarding species identification (molecular and morphological) and plant species composition are available in the Supplementary Materials. All fungal specimens were dried and preserved in the key laboratory of adaptation and evolution of plateau biota, Chinese Academic of Sciences.

### Soil sample collection

For each of the three types of FRs, a standard circular or arc-shaped FR was selected as the subject of study. Within each FR type, five rings were chosen, resulting in a total of 15 FRs. Six soil samples were collected from each FR across different zones (IN, ON, OTU) and depths (0–10, 10–20 cm), yielding a total of 90 samples. The uniform 20 cm interval between adjacent zones was determined based on our previous research showing that *Floccularia luteovirens* on the Qinghai–Tibet Plateau exhibits a fairy ring expansion rate of 0.37 m per year [31], as well as on the slower growth rate and smaller diameter (generally < 2 m) of the FRs observed in T1 and T3 in this study (Table S1). All soil samples were collected and filtered through a sieve (0.9 mm) to remove plant roots and stones, which were quickly preserved in liquid nitrogen for subsequent use.

### Assessment of soil physicochemical properties

The physicochemical parameters of the soil examined in this study encompassed soil pH, total phosphorus (TP), total carbon (TC), total potassium (TK), total nitrogen (TN), nitrate nitrogen (NO_3_^−^), ammonium nitrogen (NH_4_^+^), carbon to nitrogen ratio (C: N), and moisture content (MC). Soil pH was measured using a pH meter with a 1:1 soil-to-water ratio. TP, TC, TK, and TN were determined through standard wet chemistry methods involving digestion and subsequent quantification via spectrophotometry or flame photometry. Nitrate and ammonium nitrogen concentrations were measured using ion-selective electrodes and colorimetric assays, respectively. The C: N ratio was calculated based on the total carbon and nitrogen content. Moisture content was determined by drying soil samples at 105 °C to constant weight. For further details on these methods, please refer to our prior publication [26]. Plant diversity in different regions of the FR was characterized by calculating the Shannon-Wiener Index, which involved assessing the coverage of each species.

### FR fungi identification and HiSeq sequencing

Soil DNA was extracted using the FastDNA SPIN Kit for Soil (MP Biomedicals, California) according to the provided instructions. The samples were subsequently dispatched to Novogene Co., Ltd. in Beijing, China, for sequencing on a Hiseq-PE250 platform. The primers 515 F (5′-GTG CCA GCM GCC GCG GTA A-3′) [32] and 907R (5′-CCG TCA ATT CCT TTG AGT TT-3′) [33] were chosen for sequencing the V4-V5 region of the 16S rRNA, and the primers ITS1F (5′-CTT GGT CAT TTA GAG GAA GTA A-3′) and ITS2R (5′- TCC TCC GCT TAT TGA TAT GC-3′) [34] were chosen for sequencing the ITS1 region of the ITS (internal transcribed space). PCR products were purified using EasyPure Quick Gel Extraction Kits (TransGen Biotech, Beijing, China), and libraries were constructed with the TruSeq™ DNA Sample Prep Kit (Illumina, San Diego, CA). Sequencing data were submitted to GenBank with the accession numbers SAMN49041671-SAMN49041760 (for bacterial sequences) and SAMN49511010 -SAMN49511099 (for fungal sequences).

To identify FR fungi, we selected mature fungal fruiting bodies from each FR. DNA from fungal fruit bodies was extracted utilizing the CTAB method, and amplified for ITS fragments using primers ITS4 (5′-TCC TCC GCT TAT TGA TAT GC-3′) and ITS5 (5′-GGA AGT AAA AGT CGT AAC AAG G-3′) [35]. After sequencing, the sequences were compared at NCBI (https://www.ncbi.nlm.nih.gov/) for species identification. All sequences were uploaded to GenBank under accession numbers PV867262-PV867276. Based on the ITS BLAST alignment results, we further constructed a phylogenetic tree for the fungi. Sequences were aligned using MAFFT (v.7.526) with default parameters for multiple sequence alignment [36]. The phylogenetic tree was then constructed using FastTree software (v.2.2) [37] to infer the maximum likelihood (ML) tree, which was used to evaluate the phylogenetic relationships among fungal communities from different fairy ring types. The tree was constructed using the default model settings (JTT + CAT), and 1000 bootstrap replications were performed to assess the reliability of the tree. Due to the high similarity between T2 fungi and *A. campestris*, the construction of its phylogenetic tree was based entirely on sequences from fungi within the *Agaricus* genus. T1 and T3 fungi showed low similarity to existing data in the NCBI database (exhibiting the highest similarity to fungi from *Agrocybe* and *Clitocybe*). Therefore, corresponding sequences from Strophariaceae and Clitocybaceae species were introduced into their phylogenetic trees, respectively, to determine their phylogenetic positions. For Strophariaceae sequences, we prioritized published relevant sequences. *Agaricus* sequences primarily referenced the taxonomic study on Chinese *Agaricus* sections by An-Qi Liu et al., [38]. Clitocybaceae species sequences mainly referenced Zheng-Mi He’s phylogenetic study on the family Clitocybaceae [39].

### Bioinformatics

We analyzed the sequencing data using the Quantitative Insights into Microbial Ecology (QIIME 2 1.9.1) pipeline (https://qiime2.org/) [40]. Using the FLASH software [40], filter raw sequences with average quality scores of < 20 or read lengths of < 200 bp. The shortlisted high-quality sequences were denoised into amplicon sequence variants (ASVs) using the DADA2 pipeline [41]. The taxonomy of ASVs was analyzed using the RDP Classifier against the SILV*A* (138.2) database for bacteria and UNITE (version 9.0) for fungi. ASVs that did not belong to bacteria or fungi were removed before analysis [42]. As we did not identify species linked to FR fungus in our species annotation results, we constructed a local BLAST database for comparison using the ITS full-length sequences already obtained for the above fungi. The ASVs with the lowest *E*-values were considered to be the ASVs of the corresponding FR fungi (ASV114 for *Agrocybe* sp., ASV1146 for *A. campestris*, and ASV86 for *Clitocybe* sp.). In addition, we also extracted the sequences of these ASVs and compared them in NCBI; the results were consistent with those in our local database. To analyze the correlation between the ASVs of FR fungi and those of other microorganisms, we calculated Spearman correlations using the psych package (v.2.5.3) in R [43]. The significance of the difference in α-diversity and relative abundance of soil microbial communities across different zones and depths of FRs was measured using the Kruskal-Wallis test (with the Benjamini-Hochberg false discovery rate (FDR) correction) calculated by STAMP (v.2.1.3) software [44]. The beta diversity of the bacterial and fungal communities was calculated using non-metric multidimensional scaling (NMDS) based on Bray–Curtis distance matrices in the “vegan” package [45]. The LEfSe (Linear Discriminant Analysis Effect Size) analysis was performed to identify microbial taxa with significant abundance differences across groups. Effect size quantification by linear discriminant analysis (LDA) ranked significant taxa based on their group-discriminatory power, with features achieving LDA score > 2.0 (logarithmic scale) retained as biomarkers, the analysis was implemented in R 4.3.0 with the microeco package (v0.17.0) [46]. The differential abundance analysis of ASVs was performed using DESeq2 [47] in R, with a log2 fold change threshold of ± 1 and significance determined by an adjusted p-value (padj < 0.05).

To control for seasonal environmental variation associated with asynchronous sampling among fungal ring types, multiple climatic and soil environmental variables were explicitly included as covariates in the statistical analyses. These variables included air temperature, air humidity, precipitation, soil temperature, and soil moisture, all measured at the time of sampling which obtained from Open-Meteo database (https://open-meteo.com). Prior to model construction, we performed a variable selection process to avoid multicollinearity and ensure biological relevance. Meteorological variables (air temperature and precipitation) were excluded from the final models because they were strongly correlated with the edaphic factors. We prioritized soil temperature and soil moisture as covariates because they represent the direct micro-environmental conditions experienced by the soil microbiome. These edaphic factors offer a more proximal and physiologically relevant explanation for seasonal variations compared to indirect atmospheric measures.” Alpha diversity (Shannon index) was analyzed using linear mixed-effects models implemented in the lme4 and lmerTest packages in R [48]. Analysis of Variance (PERMANOVA) using the adonis2 function in the vegan package (999 permutations) [45]. Prior to analysis, all environmental variables were standardized using Z‐score transformation to improve model convergence and comparability of effect sizes. Climatic and soil environmental variables were included as continuous covariates to statistically control for seasonal environmental fluctuations. Fairy ring types was included as a random intercept to account for the non‐independence of samples collected within the same fungal ring. Model significance was assessed using type III ANOVA with Satterthwaite’s approximation for degrees of freedom. Post hoc comparisons among zones were conducted using estimated marginal means (EMMs), representing group means adjusted for environmental covariates.

The Mantel test was used to measure the relationships between the dissimilarity of microbial communities in different regions of the FRs (based on Bray-Curtis community dissimilarity) and soil physico-chemical properties, and plant communities (measured using the Shannon-Wiener Index). Venn diagrams analyzed bacteria unique to and shared by each component at the online platform Evenn (http://www.ehbio.com/test/venn/#/) [49]. Microbial co-occurrence networks were analyzed by using the R package meconetcomp based on SparCC analysis (SparCC correlation coefficient > 0.5 and a significance level of *p* > 0.05) [50]. Parameters that measure the network’s topology include the total number of nodes, total number of links, average degree, average path length, centralization betweenness centrality, and centralization degree centrality. To identify potential core microbes, we introduced the concept of keystone nodes. The within-module connectivity (*Z*_i_) and among-module connectivity (*P*_i_) are computed for each node, and nodes are considered to be module hubs when *Z*_i_ ≥ 2.5, *P*_i_ < 0.62, and considered to be connectors, when *Z*_i_ ≥ 2.5, *P*_i_ ≥ 0.62 nodes are considered network hubs (keystone nodes).

## Results

BLAST alignment results indicate that the T2-type FR fungi exhibit the highest similarity to *A. campestris*, a conclusion further supported by phylogenetic tree and morphological analysis (Figs. S3, S4 and supplementary materials). For the ITS sequences of the T1 and T3-type FR fungi, BLAST alignment results showed relatively low similarity (approximately 80%) to known species in the database (Figs. S2, S6). Further morphological evidence supported that the T1-type FR fungus (Fig. S1 and supplementary materials) belongs to the genus *Agrocybe*, while the T3-type FR fungus (Fig. S5 and supplementary materials) belongs to the genus *Clitocybe*.

**Fig. 2:**
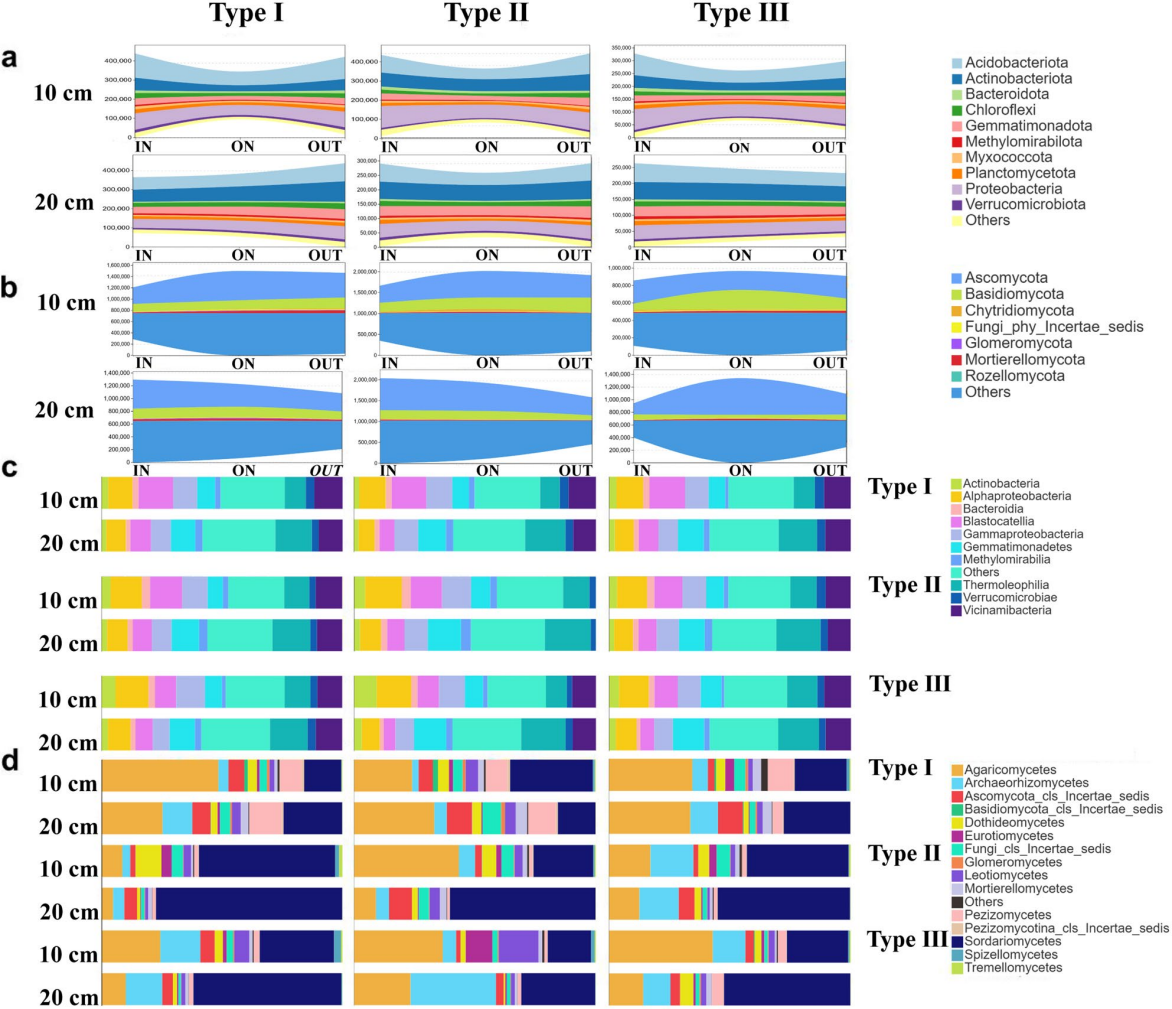
The read numbers and composition of bacterial (**a**) and fungal (**b**) at the phylum level for different FRs, zones, and soil depths. Stacked histograms of composition of bacterial (**c**) and fungal (**d**) colonies at the class level for different FRs, zones, and soil depths

This study involved 90 samples, yielding a total of 9,305,043 bacterial reads through high-throughput sequencing for three types of FRs: 3,748,181 reads for T1, 2,932,720 reads for T2, and 2,624,142 reads for T3 (Fig. 2a). A total of 7,442 bacterial ASVs were obtained based on these data. In FRs T1, T2, and T3, 6,057, 6,571, and 5,759 ASVs were identified, respectively. Most of the On zones of various fairy types and depths exhibit the lowest number of reads and ASVs in comparison to other zones (Fig. 2a). However, we did not find significant differences in the number of reads and ASVs between the ON and the other zones. We identified a total of 4,093 ASVs in the soil fungal community, comprising T1 (2,827), T2 (2,803), and T3 (2,419) from 17,537,624 reads (Fig. 2b). Unlike the case of bacterial communities, the On zones have the highest number of fungal reads and ASVs in some types of FRs. For example, the On zones of type T3 have the highest number of fungal reads and ASVs compared to other zones; however, this difference is also not significant. In addition, the percentage of unique bacterial and fungal ASVs was less in the surface soil than in the deeper soil layer. At the same time, we found that in most cases, sub-surface soils shared a greater proportion of fungal and bacterial ASVs between different zones, the only exception being T3 (Figs. S4 and S5).

In terms of species composition of the bacterial community, the dominant phylum in different soil layers and FRs were Proteobacteria, Gemmatimonadetes, Actinobacteria and Acidobacteria (Fig. 1a). At the bacterial class level, the bacterial community’s distribution showed a homogenized pattern (relative abundance of bacterial taxa that did not reside in an overwhelming majority) (Fig. 2a). For the species composition of the fungal community, the dominant fungal phyla were Ascomycota and Basidiomycota, and the relative abundance of Basidiomycota was higher than that of deeper soils in the majority of surface soils (Fig. 1b). At the level of fungal class, the dominant classes were Agaricomycetes, Sordariomycetes, and Archaeorhizomycetes. The relative abundance of Agaricomycetes in the surface soil was also higher than that of deeper soil in most samples (Fig. 2b).

**Fig. 3:**
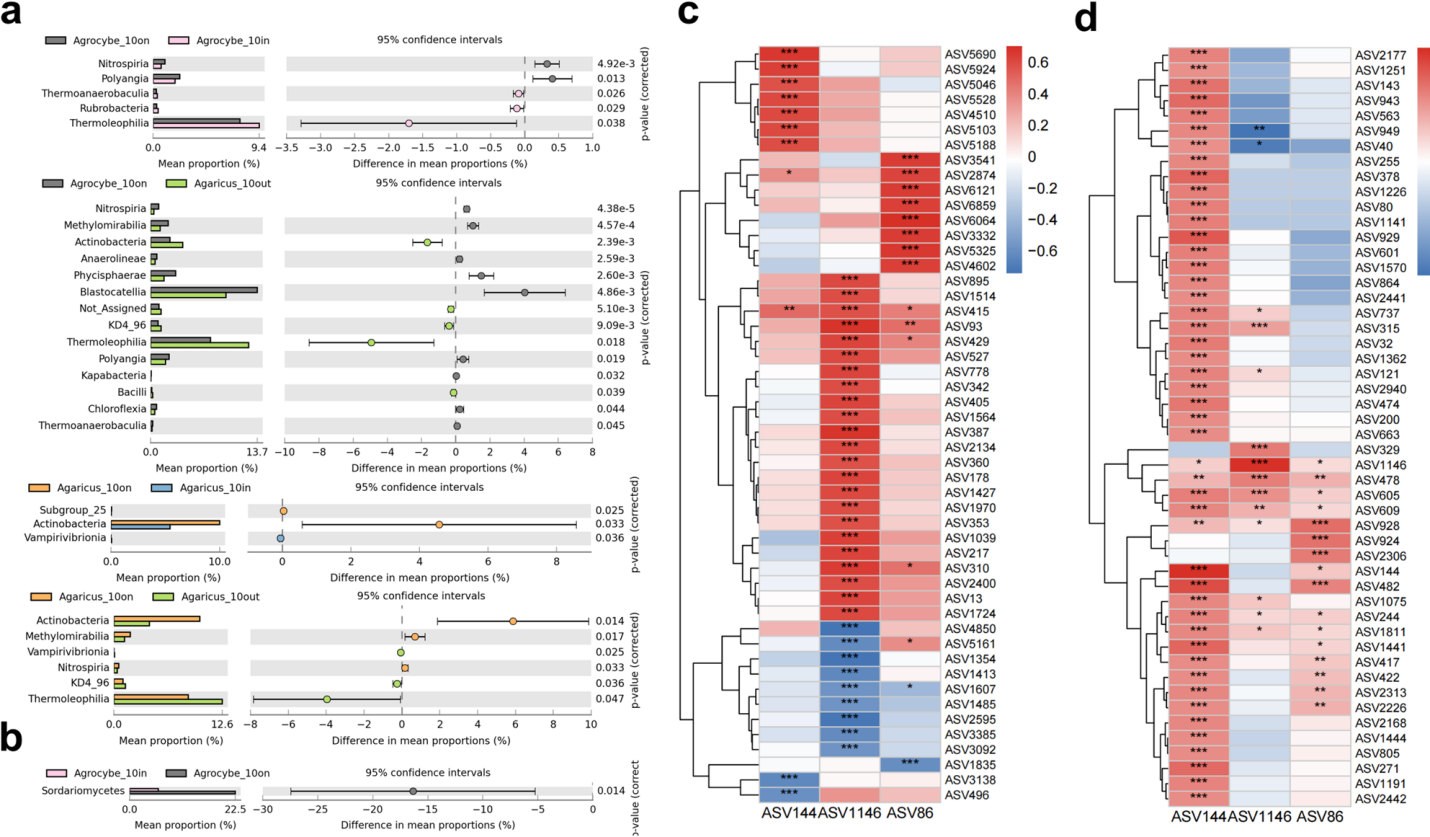
Analysis of differences at the Class level (**a**, bacteria;** b**, fungi) in different zones of surface soil in different FRs based on the Kruskal-Wallis test (using the Benjamini-Hochberg false discovery rate (FDR) correction). Heatmap showing the correlation of FR fungal ASVs (ASV144 for Type I, ASV1146 for Type II, and ASV86 for Type III FR) with bacterial (**c**) and fungal (**d**) ASVs (Spearman’s correlation analysis, only the top 50 ASVs in relative abundance are shown).

At the bacterial phylum level, no bacterial phylum with significant differences (*p* < 0.05) in relative abundance in different zones of the same FR was found based on the Kruskal-Wallis test. At the bacterial class level, we detected more differences. First, We found that all types of FRs except T3 had significantly different bacterial classes between different soil layers and zones; for example, we found that the relative abundance of Actinobacteria was significantly higher in the ON zone than in the other zones in T2 in both soil layers (Fig. 3a), we also found that the relative abundance of Nitrospiria and methylomirabilia in the surface soil of the T1 was significantly higher in the ON zone than in the OUT and the IN zones, respectively (Fig. 3a). In addition, we found more bacterial classes with significant relative abundance differences when comparing different zones of the FR in sub-surface soil (Fig. S9). At the fungal class level, in the surface soil, we found substantial differences in the relative abundance of Sordariomycetes in the ON zone of T1 compared to the other zones (Fig. 3b). We found more differences at the fungal class level in different zones in the sub-surface soil (Fig. S10). The LEfSe analysis indicated that the bacterial ASVs enriched in the ON zones of T1 included Thermomicrobia, Proteobacteria, Nitrospiria, Phycisphaerae, Gemmatimonadetes, Gammaproteobacteria, and Betaproteobacteria, while T2 predominantly enriched Thermoleophilia, Proteobacteria, Alphaproteobacteria, and Actinobacteria. Simultaneously, in T3, the enriched groups are Thermoleophilia, Proteobacteria, Gemmatimonadetes, and Actinobacteria. Furthermore, we discovered that the predominant fungal ASVs enriched in the ON zone of the T1 and T2 were Agaricomycetes and Dothideomycetes. Conversely, in T3, we did not identify any fungal class enriched in the ON zone (supplementary materials). The DESeq2 analysis results were largely consistent with those from the Lefse analysis. We detected enrichment of Thermoleophilia, Actinobacteria, and other taxa in the on-zone samples across all three types of FRs. Additionally, we observed specific enrichments of Nitrospiria in T1 and Bacilli in T2 (supplementary materials). Additionally, we found that the fungal taxa enriched in the ON zone across the various types of FRs were primarily Agaricomycetes and Dothideomycetes (supplementary materials).

Regarding microbiological alpha diversity (Shannon index) and β-diversity, we observed that nearly all significant differences were restricted to variations between soil layers (Fig. S11a, b). The examination of fungal β-diversity revealed that the discrepancies were predominantly in the T2 FR; specifically, the ON zone of the surface soil exhibited considerable variances compared to the other zones. Furthermore, we observed that the β-diversity of bacterial (Fig. S12a) and fungal (Fig. S12b) communities exhibited substantial differences between T2 and the other types, whereas the α-diversity showed no significant variation.

We identified the ASVs of the FR fungi for each type of FR, with ASV114, ASV1146, and ASV86 corresponding to the T1, T2, and T3 types of FRs, respectively. Spearman’s correlation analysis revealed that the ASVs of these FR fungi were significantly correlated with certain soil microorganisms in the ON region of their respective FRs and that more bacteria than fungi were found to be significantly correlated (Supplementary Materials). For example, T1, T2, and T3 were significantly associated with 289, 690, and 461 bacterial ASVs (Fig. 3c ) and 675, 305, and 287 fungal ASVs (Fig. 3d), respectively. Bacterial ASVs that were significantly and positively correlated with ASV114 (T1) ASV1146 (T2) and ASV86 (T3) were mainly Proteobacteria (35, 159 and 76, respectively) (Fig. 3c). The fungi with the highest number of ASVs that were significantly positively correlated with ASV114 (T1) ASV1146 (T2) and ASV86 (T3) were Dothideomycetes and Sordariomycetes (Fig. 3d). In contrast, the fungi with the highest number of significant negative correlations were Agaricomycetes (Fig. 3d) (Supplementary Materials).

**Fig. 4:**
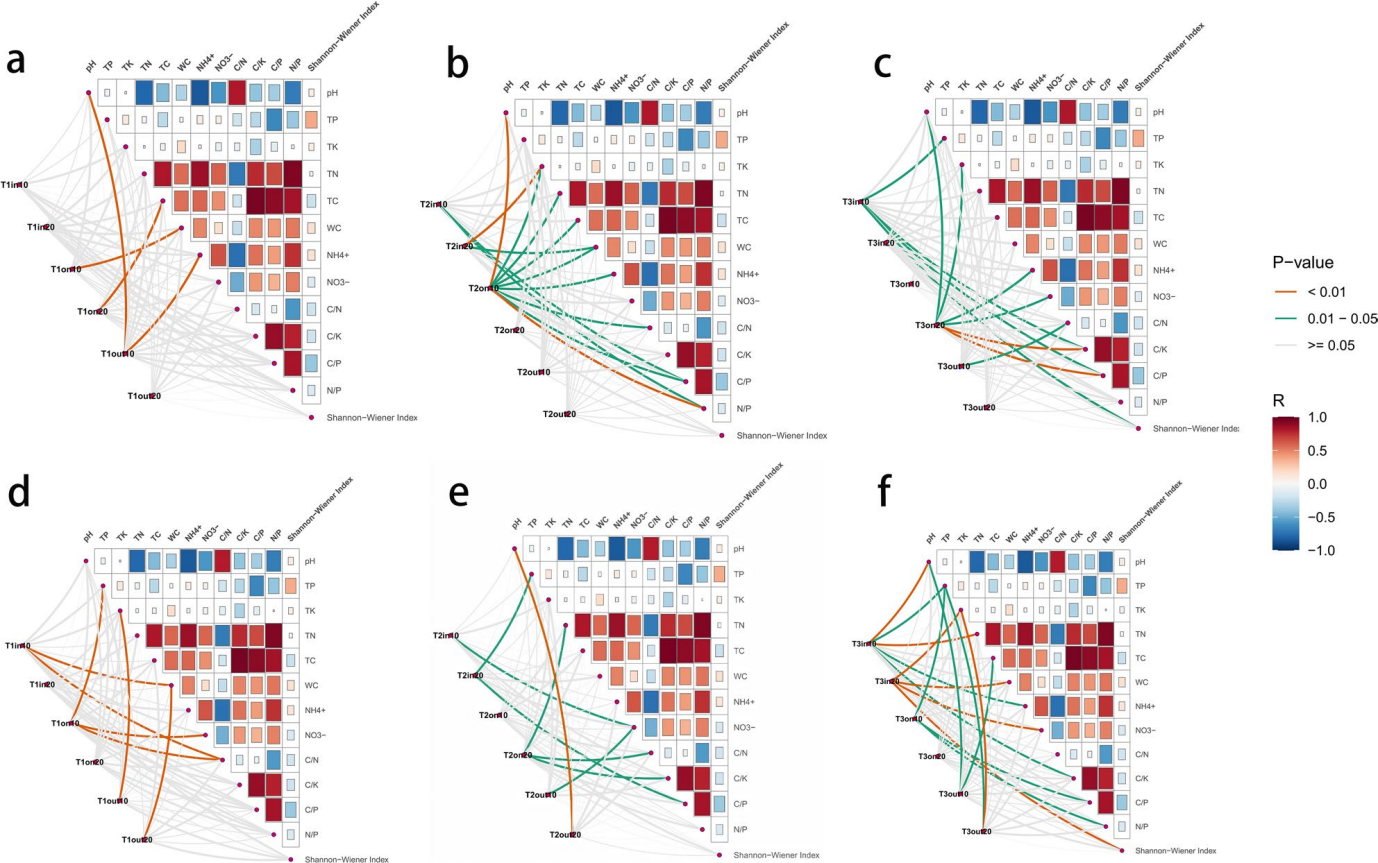
Correlation between soil microbial communities and soil physicochemical properties and plant diversity indices based on the mantel test (red line indicates significant correlation *p* < 0.05, **a** and** d** show the soil bacterial and fungal communities of the type I FR,** b** and** e** show the soil bacterial and fungal communities of the type II FR, and** c** and** f** show the soil bacterial and fungal communities of the type III FR)

After statistically controlling for seasonal environmental fluctuations using LMMs, we found that neither soil temperature (*p* = 0.887 (bacterial community), *p* = 0.261(fungal community)) nor moisture (*p* = 0.393 (bacterial community), *p* = 0.187(fungal community)) had a significant effect on the Shannon diversity index. However, soil depth significantly influenced alpha diversity (*p* = 0.018 (bacterial community), *p* = 0.0003(fungal community) (Supplementary Materials). In contrast to alpha diversity, the PERMANOVA results revealed that seasonal environmental factors significantly shifted the microbial community structure. However, even after partitioning out the variance attributed to these seasonal edaphic factors, the biological effects remained robust both in the soil bacterial and fungal communities. The fairy ring type, soil deepth, and zonal position continued to significantly explain the variation in microbial community composition. Furthermore, soil depth remained a strong predictor of community structure (*p* = 0.001), independent of seasonal changes (Supplementary Materials).The analysis of the correlation between soil physicochemical properties and soil microbial communities in different types of FRs revealed that distinct soil physicochemical properties influenced bacterial and fungal communities from various FR types to varying degrees. In the T1, the bacterial community in the surface soil of the ON zones was only significantly influenced by WC (Fig. 4a). We found that the soil bacterial community in the surface ON zone of T2 was significantly influenced by the most environmental factors, whereas the subsurface soil bacterial community showed no significant environmental influences. Conversely, we did not identify any environmental factors significantly affecting the soil fungal community in the surface ON zone of T2 (Fig. 4b and e). Within the T3 ON zone, C/P, C/K, NO_3_^−^, NH_4_^+^, TK, and pH were key drivers for the subsurface bacterial community, while C/P, N/P, and TP significantly determined the surface fungal community structure (Fig. 4c and f).

**Fig. 5:**
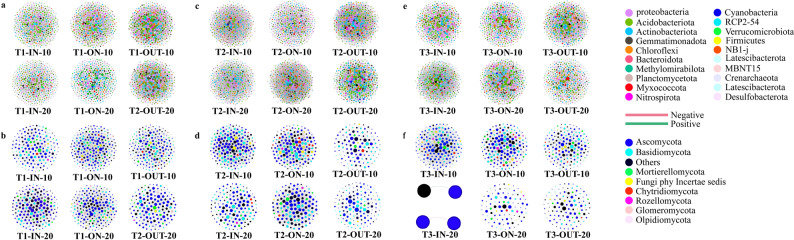
Intra-kingdom co-occurrence networks. Node size indicates the degree of connection. Edge color represent positive and negative correlations.** a**,** c**, and** e** are the co-occurrence networks of the bacterial communities of the T1, T2, and T3 types of FRs, respectively.** b**,** d**, and** f** are the co-occurrence networks of the bacterial communities of the T1, T2, and T3 types of FRs, respectively

**Fig. 6:**
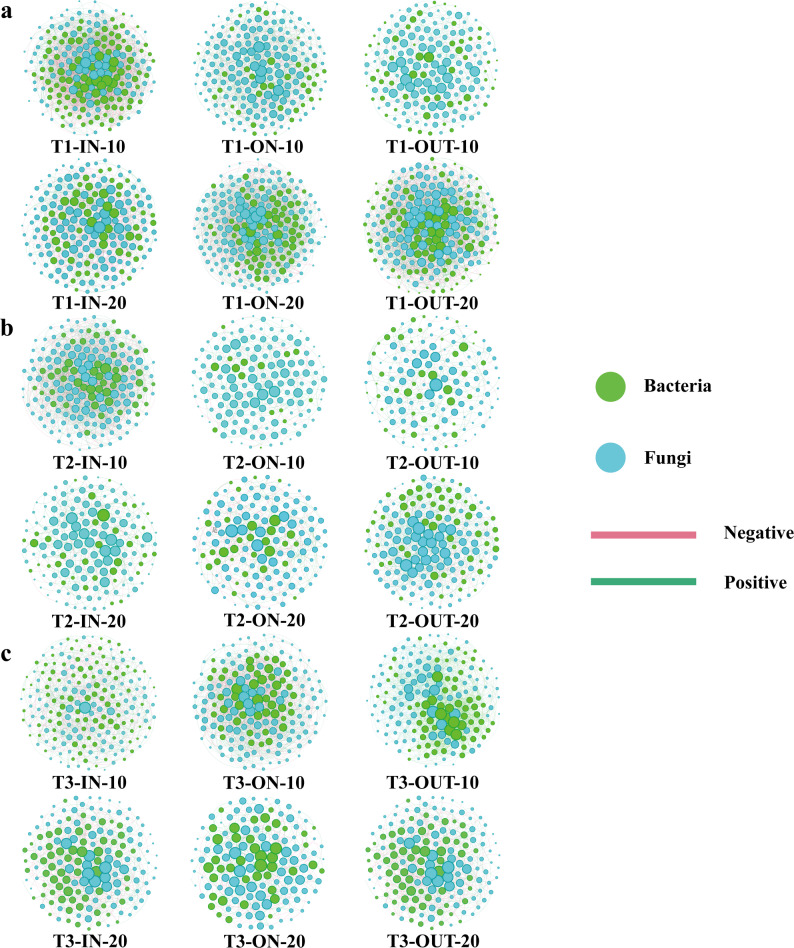
Inter-kingdom co-occurrence networks of three types of FRs (**a**, T1 type FR;** b**, T2 type FR;** c**, T3 type FR). Node size indicates the degree of connection. Edge color represents positive and negative correlations

The analysis of the microbial co-occurrence network indicated that in the bacterial communities, density of the network, the clustering coefficient, and the centralization of the network were almost always highest in both T1 (Fig. 5a and Table S2) and T3 (Fig. 5e and Table S2), while the above metrics were highest in the ON zone of the subsurface soil in T2 (Fig. 5c and Table S2). The network hubs in the ON zone exhibit the following: T1 is mostly constituted of Alphaproteobacteria, Nitrospiria, and Thermoleophilia (Fig. S13a and Supplementary Materials); T2 is primarily made of Alphaproteobacteria and Thermoleophilia (Fig. S13b and Supplementary Materials); and Actinobacteriota chiefly represents T3 (Fig. S13c and Supplementary Materials). The highest number of nodes was observed in the ON zone across almost all fungal co-occurrence networks, with lower counts in the subsurface compared to subsurface soil. Most network parameters were maximized in the IN zone for T1 (Fig. 5b and Table S3) and T3 (Fig. 5d and Table S3). All network parameters exhibited a decreasing trend as soil depth increased. The sole exception is the On zone of T2 (Fig. 5c and Supplementary Materials), characterized by increased network density, centralization of the co-occurrence network, and hubs in both networks and modules in subsurface soils (Fig. S13 and Supplementary Materials). Bacteria-fungi and fungi-fungi interactions were predominant in nearly all sample. In the surface soil of ON zones, there were greater proportions of bacteria-fungi and fungi-fungi interactions compared to bacteria-bacteria interaction. For instance, in the T2, the bacteria-fungi and fungi-fungi interactions in the surface soil of the ON zone constituted 99.0% (Fig. 6b and Table S4), whereas in the IN zone, they were below 90% (Fig. 6b and Table S4). In surface soil of IN zone of T1 and subsurface soil of IN zone of T3, positive edges predominated in the interactions; however, negative edges were more prevalent in the remaining interactions (Fig. 6a and Table S4).

## Discussion

This study provides a comparative examination of soil microbial community structures and their environmental drivers across different zones and soil depths in three types of FRs from Tibetan alpine meadows, with a particular focus on microbe–fungus interactions within the FR zones. Our investigation revealed that the number of reads, ASVs, and alpha diversity index of bacterial communities in the ON zone of various FRs was almost the lowest in comparison to other zones. In contrast, fungal communities showed the highest read numbers and ASV richness in the ON zones. Such patterns likely reflect the continuous outward expansion of the FR mycelium, which is most active in the ON zone. We propose that fungal mycelia may preferentially utilize organic carbon sources, such as cellulose and lignin, which could limit substrate availability for certain bacterial taxa. Additionally, the rapid growth and production of secondary metabolites, such as hydrocyanic acid, by some ectomycorrhizal fungi—like *Tricholoma matsutake* [21]; and *Floccularia luteovirens* [26]—can suppress specific bacterial groups and reduce overall bacterial diversity. Furthermore, these fungi may attract antibiotic-producing bacteria, which could contribute to the homogenization of bacterial communities [19, 51]. These combined effects likely explain the reduced bacterial diversity observed in the ON zones. The dense mycelial networks of FR fungi in the ON zones may also provide substrates for other saprotrophic fungi, which utilize mycelial residues and decomposition products to expand their ecological niches [26]. The acidified environment (lower pH) in the ON region promotes fungi that are adapted to low pH (e.g., Mortierella) [17] and phosphorus-dependent taxa (e.g., Umbelopsis) [20]. The factors above collectively account for the differences in fungal diversity in the ON zone of the FR. Interestingly, we observed no significant difference in these parameters between the IN and OUT zones across the three FR types, suggesting that soil microbial communities may recover rapidly after fungal disturbance.

FR systems offer a valuable natural model for exploring fungal–microbial interactions during active mycelial expansion. Prior research indicates that fungal hyphae are capable of recruiting beneficial microorganisms and inhibiting pathogenic bacteria via targeted modulation of secretion [52, 53]. Fungal hyphae may secrete antibiotics and volatile organic compounds (VOCs) that directly inhibit pathogen growth [54], such as chitinases from *Trichoderma* spp. that degrade pathogenic fungal cell walls and 2,5-dimethoxybenzoquinone from *Penicillium* spp. that inhibits the spore germination of *Rhizoctonia solani* [55]. Fungal hyphae can also release carbon sources through efficient decomposition of organic matter (e.g., lignin, cellulose), which are preferentially utilized by saprophytic fungi, resulting in the suppression of pathogenic bacteria due to carbon and nitrogen deprivation. For example, competitive uptake of organic phosphorus by *Massilia* spp. in the AM fungal mycelium significantly reduced the survival of the pathogenic fungus *Fusarium* [56]. In our dataset, enrichment analyses highlighted distinct bacterial and fungal taxa across FR types, especially within the ON zones. Across all FR types, Proteobacteria and Thermoleophilia were consistently associated with the ON zones. The presence of FR fungi appears to favor Proteobacteria such as *Pseudomonas* and *Sphingobacterium* by liberating resources, including nitrogen and carbon sources, via the decomposition of organic materials. For example, the mycelial activity of *Calocybe gambosa* enhances soil ammonium nitrogen levels, thereby conferring a growth advantage to Proteobacteria [18]. In turn, Proteobacteria facilitate the degradation of complex organic matter by fungi, which is advantageous for mycelial expansion [57]. The relationship between Thermoleophilia and FR fungi remains unexamined, and further research is therefore needed to provide conclusive evidence. Furthermore, we discovered that various types of FRs were enriched with endemic bacterial taxa. Specifically, in T1, Phycisphaerae and Nitrospiria were mainly enriched. Phycisphaerae are involved in deleting complex organic matter (e.g., cellulose, lignin) and increasing soil carbon pool stability by secreting extracellular enzymes that promote humus formation. Its metabolites (e.g., short-chain fatty acids) provide carbon sources for fungal hyphae and indirectly support mycelial expansion [58]. As a key player in soil nitrogen cycling, Nitrospiria—particularly *Comammox Nitrospira*—dominates nitrification processes, contributing up to 70% of the overall nitrification rate, especially in weakly acidic soils. Beyond its role in nitrogen transformation, *Comammox Nitrospira* also possesses a complete cobalamin metabolic pathway that provides essential coenzymes to approximately 86.8% of cobalamin-dependent microorganisms in the soil, thereby promoting interspecies cooperation and enhancing community stability [59]. Such processes may partially explain the dominance of *Leontopodium nanum* in Type I rings, as this species is tolerant to nitrate-enriched conditions [60], However, further experiments are needed to validate this hypothesis. Moreover, it has a deeper root system that maintains stability in water and nutrient acquisition in high nitrate-N environments, thereby improving nitrate-N tolerance [61]. Together, these findings suggest that *Agrocyb* FR fungi may recruit Nitrospiria and other nitrogen-transforming taxa to facilitate mycelial expansion and indirectly influence plant community composition.

Research on FRs has historically concentrated on Type II rings because their growth-promoting zones are easily observed. Multiple mechanisms have been proposed for the formation of these promotive zones, including enhanced soil nutrient availability [10], improved soil aggregation [62], and the production of phytohormone-like compounds such as AHX and ICA [12, 13]. However, limited attention has been given to potential interactions between FR fungi and other soil microorganisms that might drive these phenomena. In recent years, numerous studies have highlighted that fungal hyphae can recruit beneficial core microorganisms to promote the growth of their own hyphae and that of plants through chemical signaling [63] and root secretion [52]. In our study, Actinobacteria, Proteobacteria, and Bacilli were enriched in the ON zones of Type II FRs. Actinobacteria contribute to soil fertility by decomposing complex organic matter and releasing soluble carbon and nutrients, while some genera (e.g., Streptomyces) enhance phosphorus availability and suppress soil-borne pathogens through antibiotic production and symbiosis with AMF [64, 65]. Proteobacteria participate in nitrogen cycling and are associated with the establishment of redox gradients at the soil–root interface [66, 67]. *Bacillus* species promote the expansion of fairy ring fungi and the proliferation of surrounding vegetation by secreting phytohormones such as indole-3-acetic acid (IAA) and extracellular hydrolases, which enhance the mineralization and bioavailability of essential nutrients like phosphorus and potassium. Furthermore, Bacillus-derived biosurfactants, such as lipopeptides, effectively alleviate soil hydrophobicity induced by dense mycelial networks, improving water infiltration to maintain the moist micro-environment necessary for mycelial advancement [68, 69]. We propose that *A. campestris* may achieve outward mycelial expansion by enriching bacterial taxa that enhance fungal growth or suppress antagonistic microbes, thereby promoting both fungal persistence and plant productivity [70, 71]. In contrast, Type III FRs showed few significant changes in bacterial composition, suggesting a weaker capacity for recruiting beneficial microbial partners, which may explain their minimal effects on vegetation. On the other hand, this study also found that the microbial communities (especially bacterial communities) in the ON zones of different FRs were affected by different soil physical and chemical properties to different degrees; similar to the results of the bacterial enrichment analyses, the bacterial community of ON zone in surface soil of T3 had the weakest correlation with the soil conversation properties, while the bacterial community in T2 had the strongest correlation with the soil conversation properties. This may reflect their ability to influence the structure of soil microbial communities through their impact on soil properties. All types of FRs were enriched with fewer fungi, mainly Agaricomycetes and Dothideomycetes. Dothideomycetes are often associated with pathogenicity and may contribute to vegetation pattern formation in the IN region of T1 [72]. The enrichment of Agaricomycetes should be attributable to the FR fungi themselves.

Microbial co-occurrence networks provide an effective framework to infer potential ecological interactions among soil microorganisms [73]. The ON zone exhibited the most complex network structure, with the highest density, clustering coefficient, and centralization in surface soils of T1 and T3, and in subsurface soils of T2. The average path length was lowest in the ON zone, indicating enhanced connectivity. Although negative correlations dominated all co-occurrence networks, the proportion of positive edges increased in the ON zone. These patterns imply a shift toward cooperative ecological strategies among bacteria under FR influence, reflecting a potential close metabolic complementarity and signal-based coordination. In contrast, fungal networks in the IN zones of Type I and II FRs were more diffuse and competitive, consistent with decentralized functioning under resource stress.

This study examined cross-kingdom co-occurrence networks to analyze the connections between bacteria and fungi. Fungal interactions predominated in all networks. Nonetheless, the ratios of bacteria-fungus and fungus-fungus interactions escalated in the ON zones. Bacteria-bacteria and fungus-fungus interactions were predominantly characterized by negative edges, indicating that microbes primarily engage in competition with one another. This corroborates our prior analysis, which suggests that FR fungal hyphae may attract or enhance the proliferation of beneficial microbies, thereby suppressing the growth of competing species. Fungal-mediated interdomain interactions are crucial in soil ecosystems, as fungi interlink plant roots via mycelial networks to establish ternary associations among fungi, bacteria, and plants [74]. The importance of such interactions is illustrated by the rising proportion of fungal-mediated interactions in the ON zones of different types of FRs in this study, where fungi may alter the local microenvironment through mycelial secretions to promote the growth of specific bacteria and, at the same time, fungal hyphae may inhibit the overexpansion of some bacteria through physical isolation or chemical signaling, while providing shelter for other bacteria [75]. In addition, we note that the co-occurrence network in the subsurface of the ON zone of T2 is more stable and that a positive correlation dominates the shift in fungal-fungal interactions, so we hypothesize that the T2 FR fungi function at a deeper depth relative to the other types.

Although we did not directly measure root or fungal hyphal biomass in this study, our primary objective was to characterize the microbial community composition and the interaction patterns between kingdoms associated with fairy ring development. While measuring belowground biomass is valuable, it primarily addresses the quantity of resources rather than the ecological relationships among microbes. Previous studies have shown that microbial stratification and differentiation in fairy rings can occur due to fungal metabolic activity and variations in soil biochemistry, even in the absence of measurable changes in root or mycelial mass. The depth-dependent microbial patterns observed in our study likely reflect these processes. Future research could benefit from directly quantifying root and hyphal biomass and employing stable isotope tracing or soil metabolomic analyses. This would help establish a clearer link between microbial community dynamics, carbon allocation, nutrient cycling, and fungal activity within fairy ring soils. A potential limitation of this study was the sampling of different fungal species across varying seasons. Our multivariate analysis, however, allowed us to disentangle these seasonal effects from the specific impacts of the fairy rings. Interestingly, while seasonal fluctuations significantly shifted the overall community composition (Beta diversity), they did not alter the species richness (Alpha diversity), suggesting a replacement of taxa rather than a loss of diversity across seasons. Most importantly, our sequential PERMANOVA models demonstrated that the unique “fingerprint” of each fungal species (FR type) and their zonal activities (Zone) exerted significant selective pressure on the microbiome, independent of the seasonal environmental baseline. This confirms that the observed fairy ring effects are intrinsic biological phenomena driven by the fungal host, rather than artifacts of sampling time or seasonal climate variations.

## Conclusion

In summary, this study provides the first integrated comparison of three FR types in Tibetan alpine meadows, linking FR fungi, soil microbial networks, and vegetation responses across spatial and depth gradients. Our results indicate that different FR types enrich distinct bacterial taxa, suggesting that they may possess the ability to selectively recruit microbes that facilitate mycelial expansion, thereby shaping soil microbial communities and influencing aboveground plant composition. These findings advance our understanding of FR-driven microbial communities and provide a foundation for future experiments to validate the mechanisms underlying these complex interactions.

## Supplementary Information

Below is the link to the electronic supplementary material.

Supplementary Material 1

Supplementary Material 2

Supplementary Material 3

## Data Availability

No datasets were generated or analysed during the current study.
