# Supplementary figures and images for: Microbial interactions mediate the fairy ring type effects on alpine meadow plant communities on the Tibetan plateau
Source: Environ Microbiome. 2026 Mar 10;21:55. doi: 10.1186/s40793-026-00873-z (PMC13088534; doi:10.1186/s40793-026-00873-z)

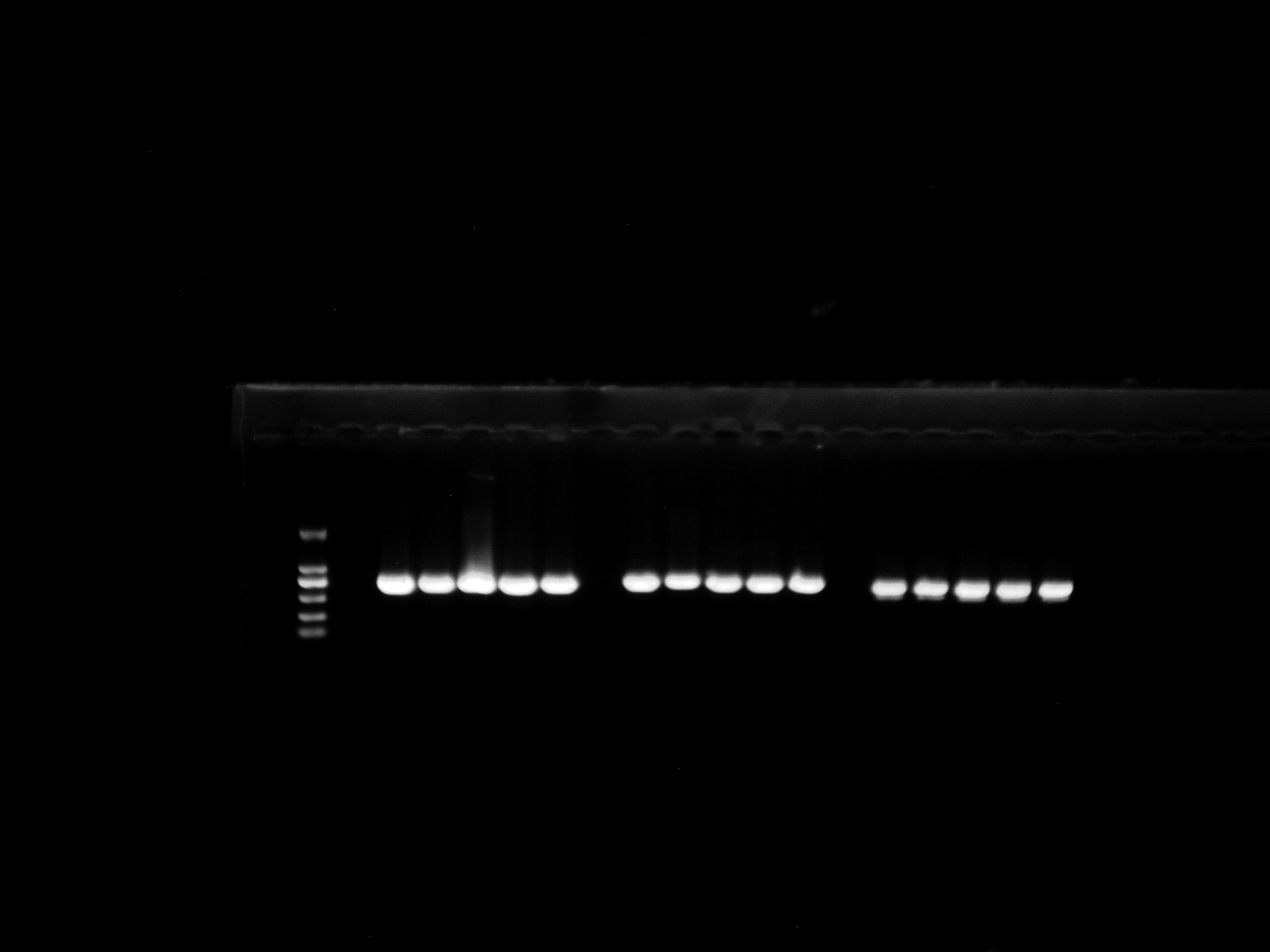

Supplement: Supplementary file 1 — Supplementary Material 1 [file 40793_2026_873_MOESM1_ESM.gif]
