# Supplementary material for: Microbial interactions mediate the fairy ring type effects on alpine meadow plant communities on the Tibetan plateau
Source: Environ Microbiome. 2026 Mar 10;21:55. doi: 10.1186/s40793-026-00873-z (PMC13088534; doi:10.1186/s40793-026-00873-z)
Supplement: Supplementary file 3 — Supplementary Material 3 [file 40793_2026_873_MOESM3_ESM.docx]

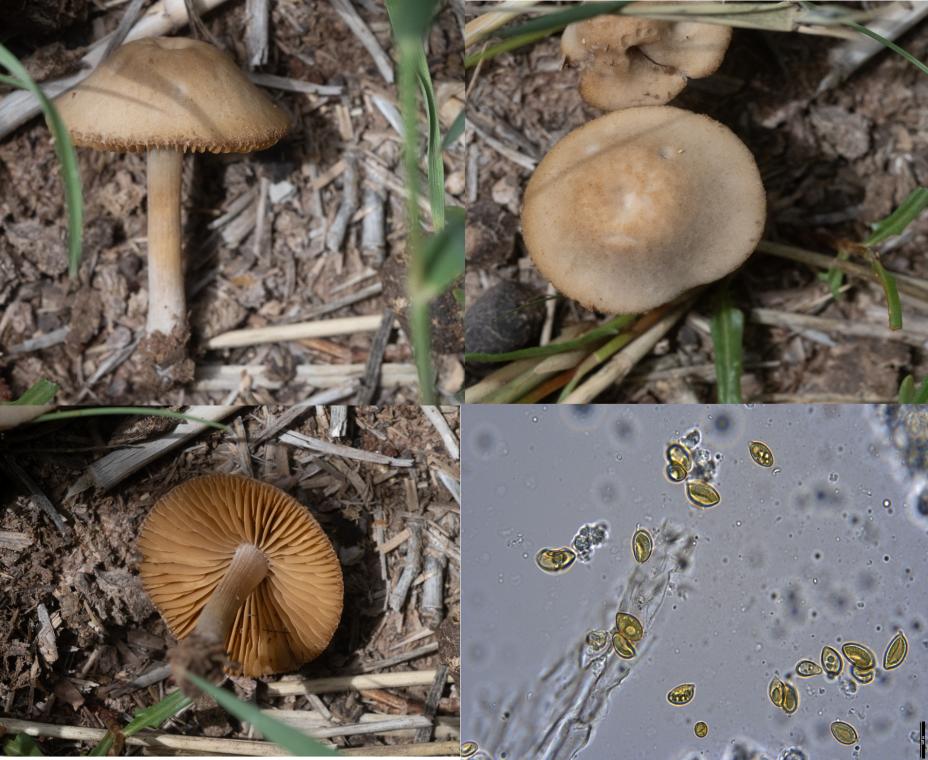
Fig.S1 Images of fruiting bodies and spores of the Type I fairy ring fungus *Agrocybe* sp.


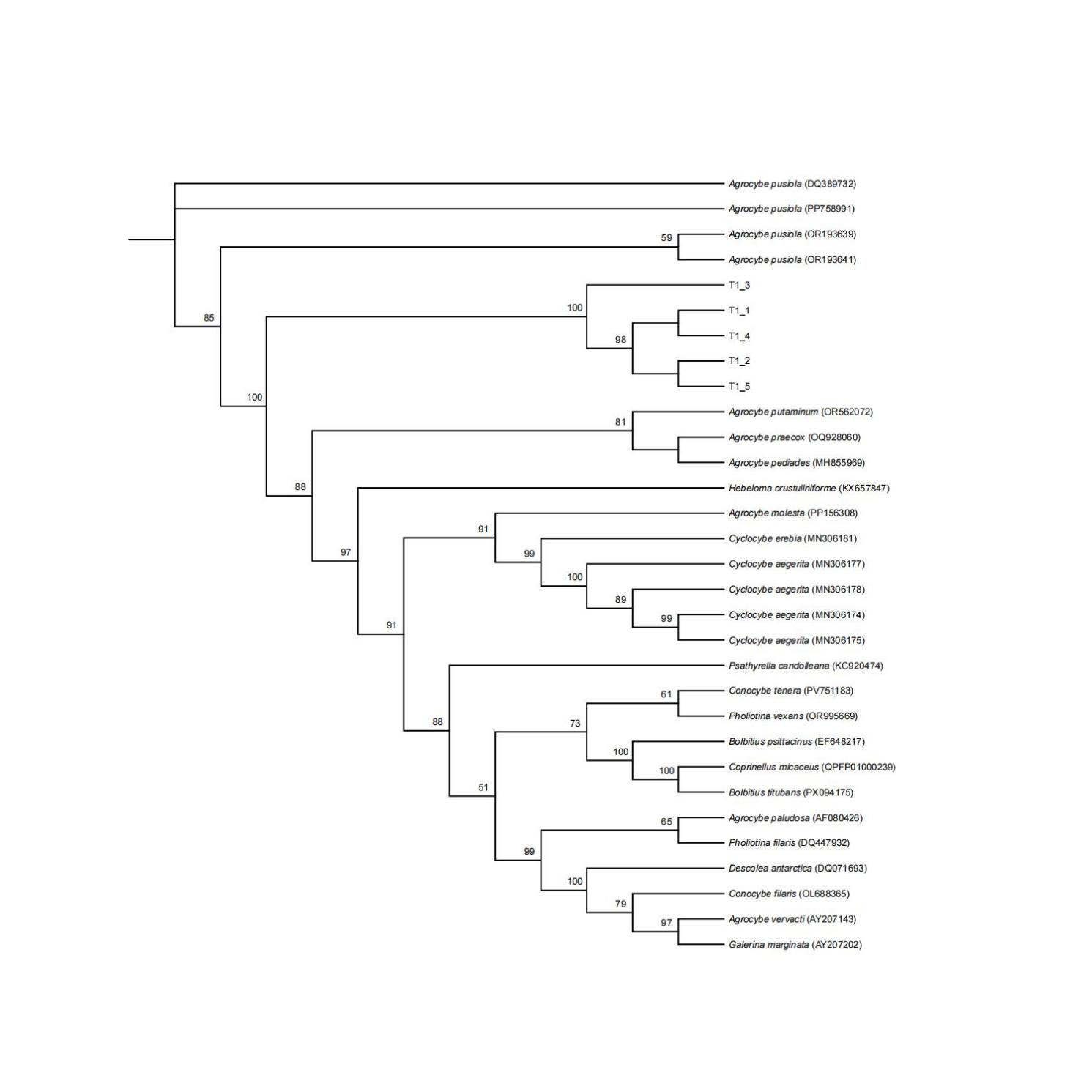


Fig.S2 A Maximum Likelihood Phylogenetic tree of fungi containing type I fairy ring fungi, including ITS sequences from fungi within the family Strophariaceae. Bootstrap values from 1000 replicates are shown as percentages at the branching points (Only values greater than 50 are displayed).


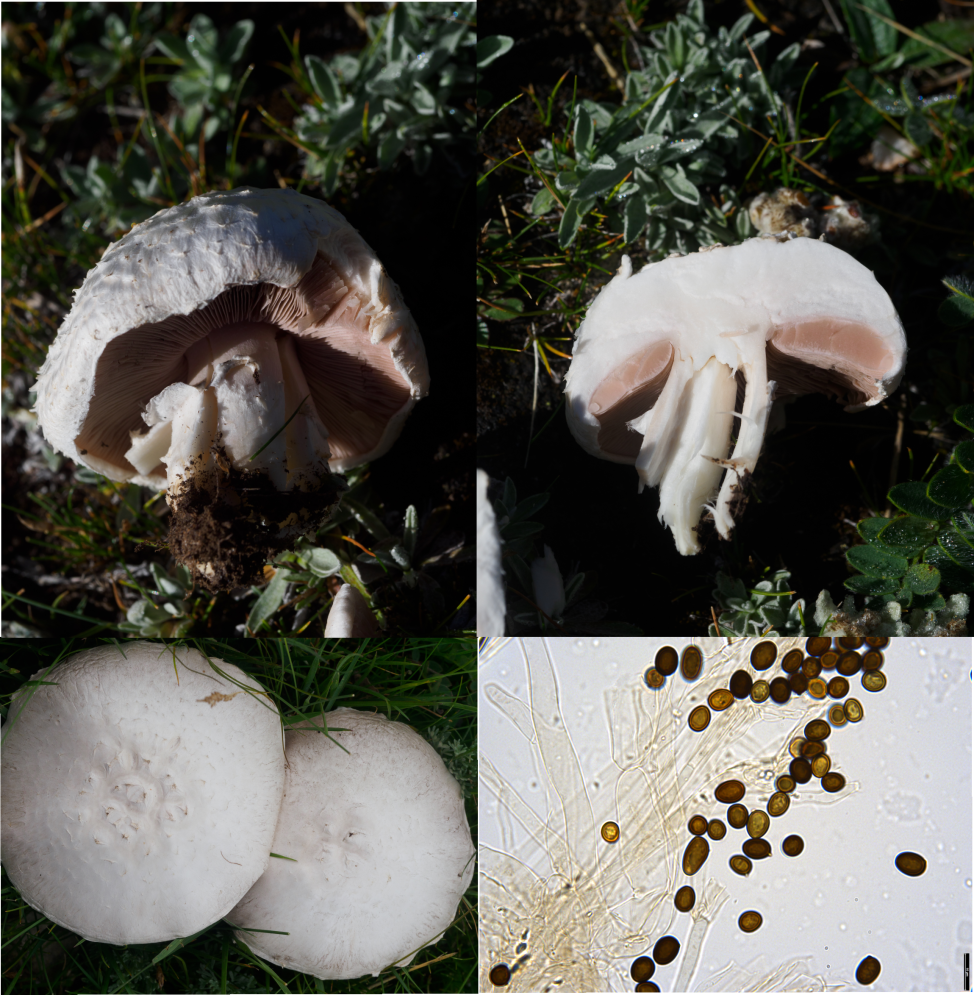
Fig.S3 Images of fruiting bodies and spores of the Type II fairy ring fungus *Agaricus campestris*


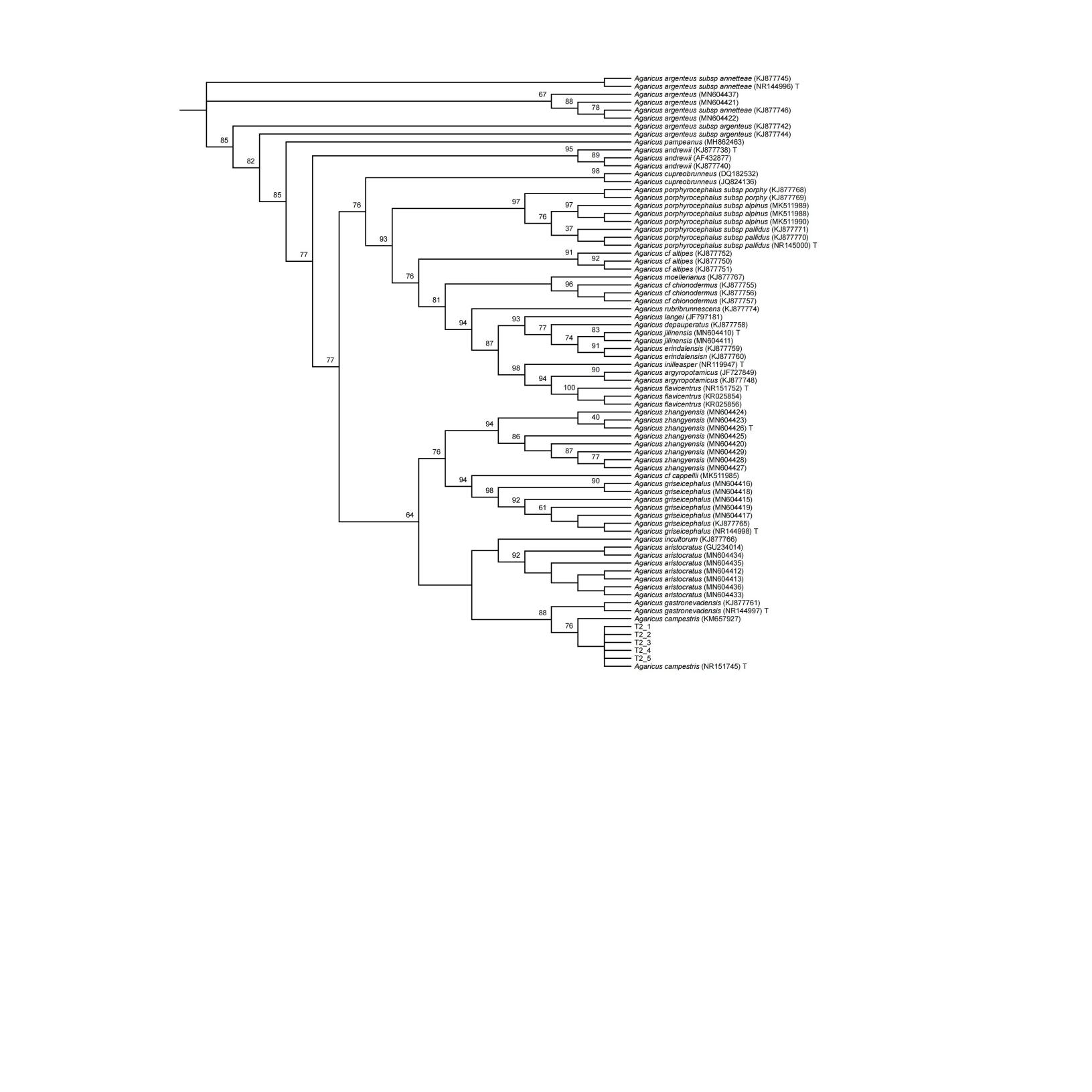


Fig.S4 A Maximum Likelihood Phylogenetic tree of fungi containing type II fairy ring fungi, including ITS sequences from fungi within the genus *Agaricus*. Bootstrap values from 1000 replicates are shown as percentages at the branching points (Only values greater than 50 are displayed).


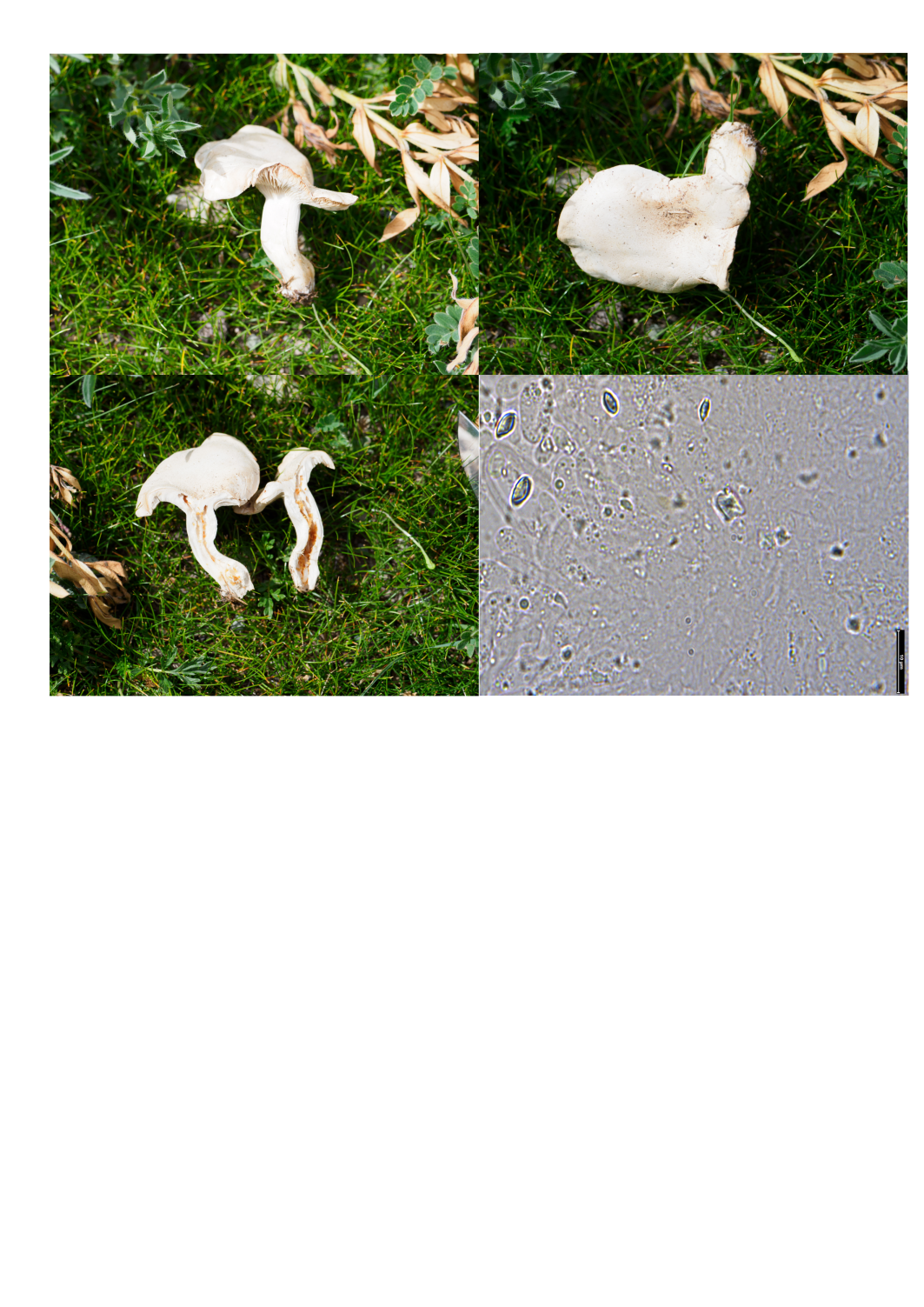
Fig.S5 Images of fruiting bodies and spores of the Type III fairy ring fungus *Clitocybe sp*.


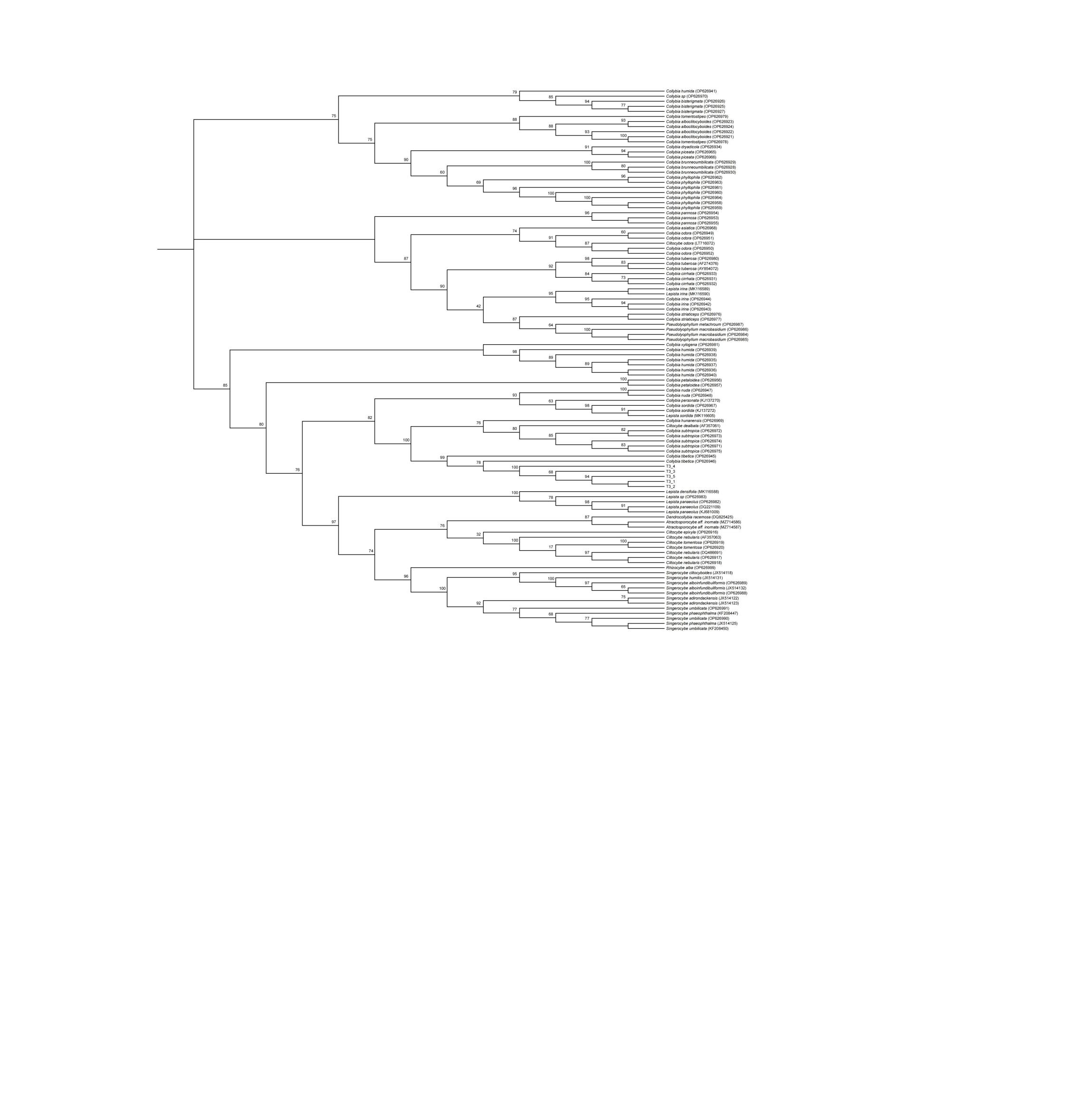


Fig.S6 A Maximum Likelihood Phylogenetic tree of fungi containing type III fairy ring fungi, including ITS sequences from fungi within the family Clitocybaceae. Bootstrap values from 1000 replicates are shown as percentages at the branching points (Only values greater than 50 are displayed).


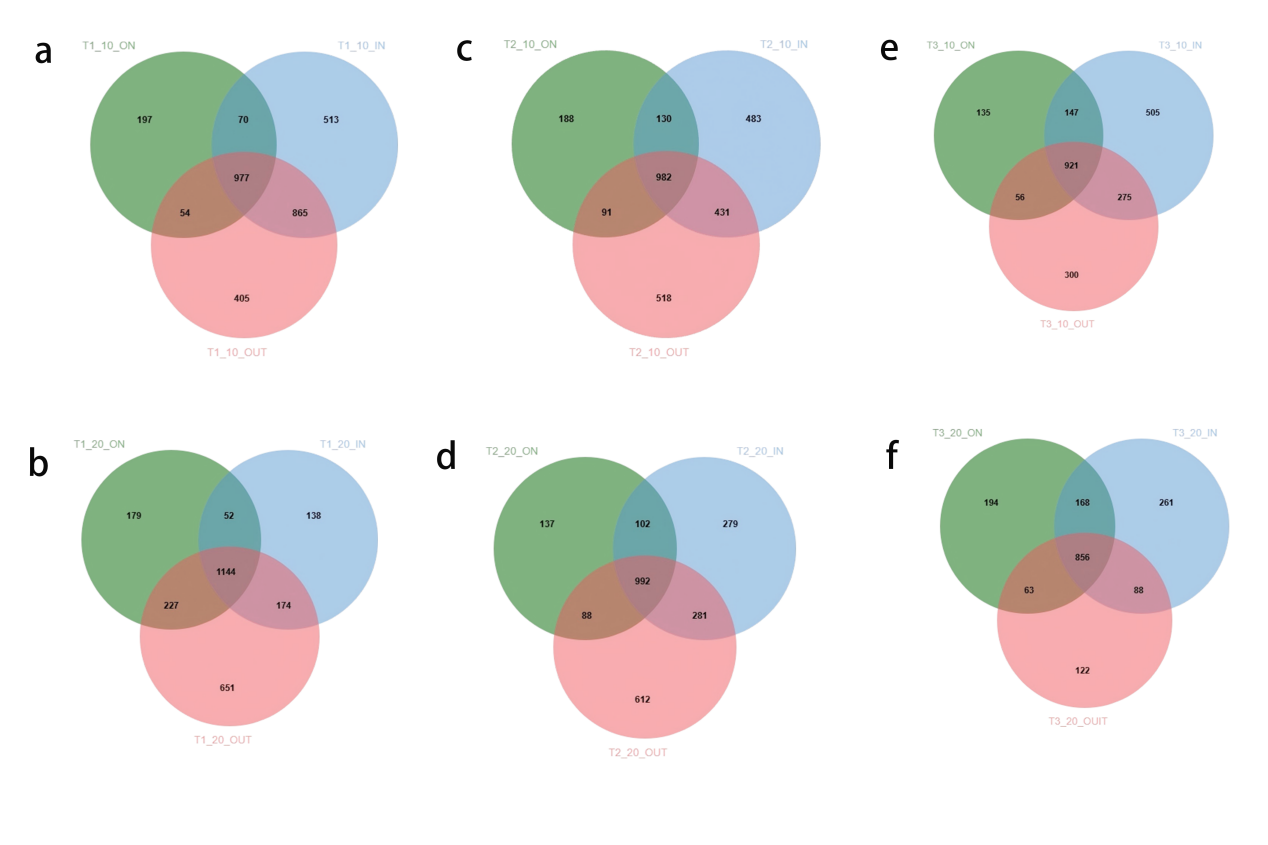


Fig.S7 Venn plots show the distribution of bacterial OTUs in different zones in the fairy ring (a and b are type I surface and sub-surface soil bacterial communities, respectively, c and d are type II surface and sub-surface soil bacterial communities, and e and f are type III surface and (sub-surface soil bacterial communities).


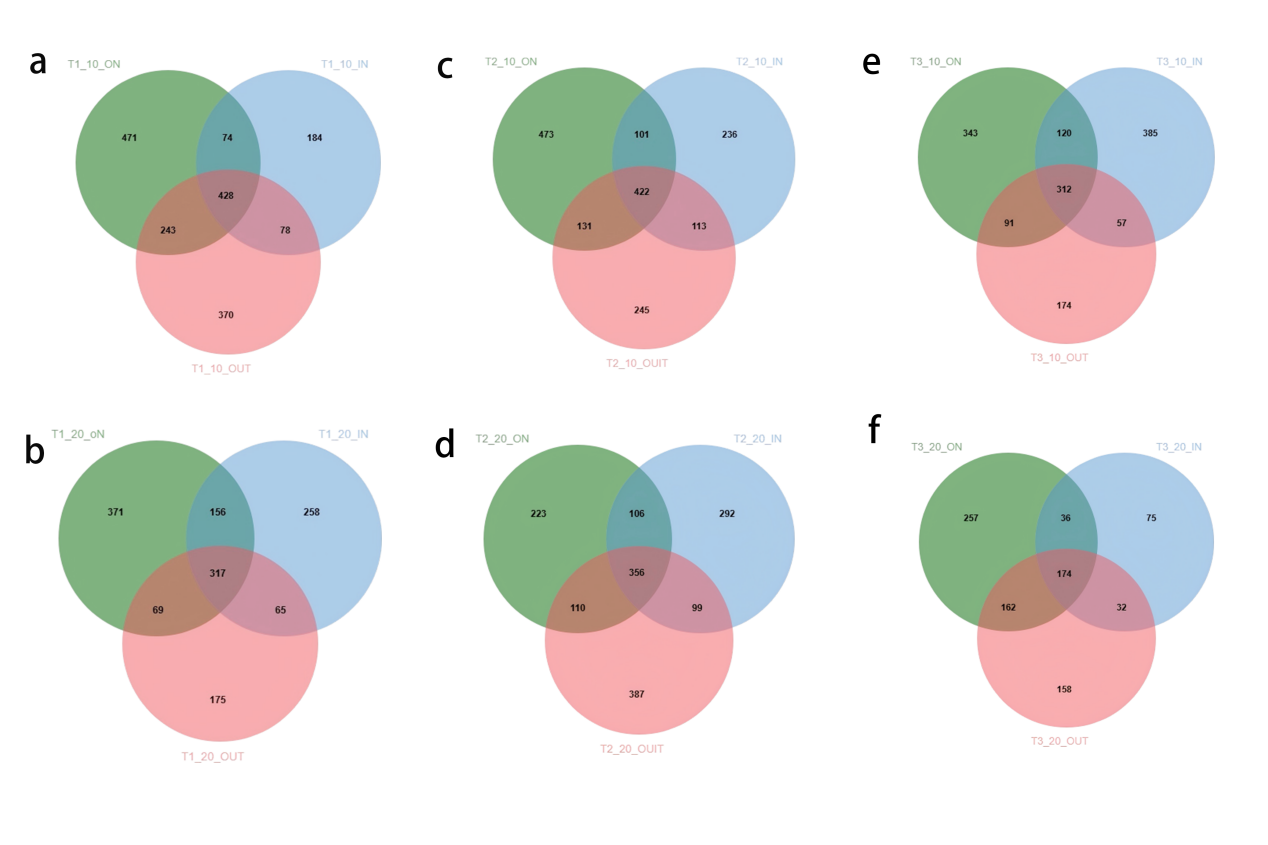


Fig.S8 Venn plots show the distribution of fungal OTUs in different zones in the fairy ring (a and b are type I surface and sub-surface soil bacterial communities, respectively, c and d are type II surface and sub-surface soil bacterial communities, and e and f are type III surface and (sub-surface soil bacterial communities).


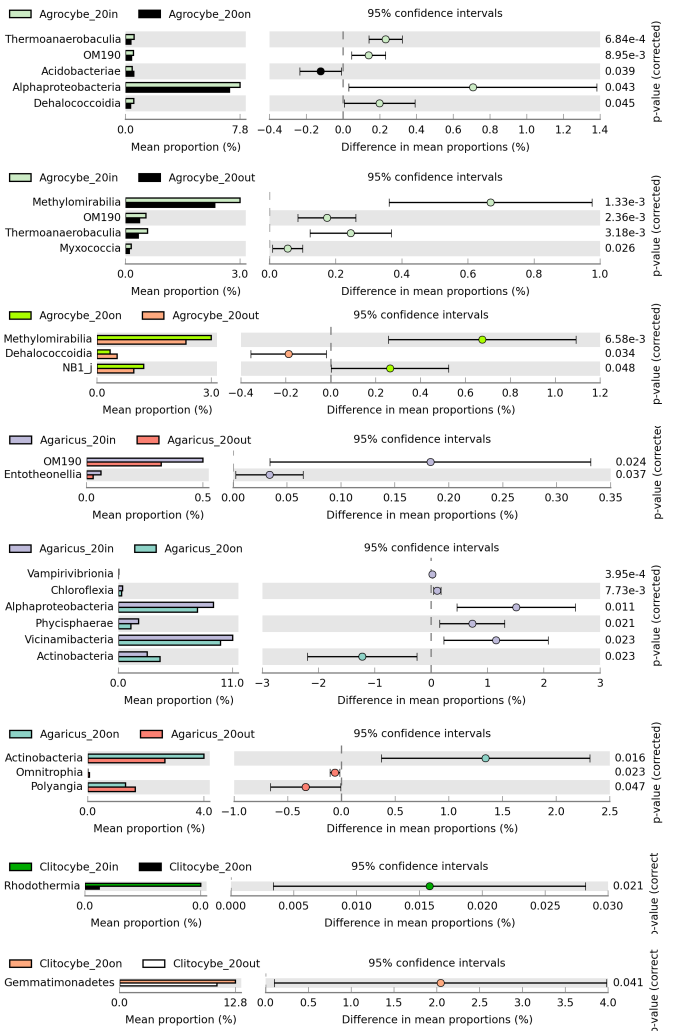


Fig.S9 Analysis of differences at the class level (bacteria) in different zones of sub-surface soil in different fairy rings based on the Kruskal-Wallis test (using the Benjamini-Hochberg false discovery rate (FDR) correction).


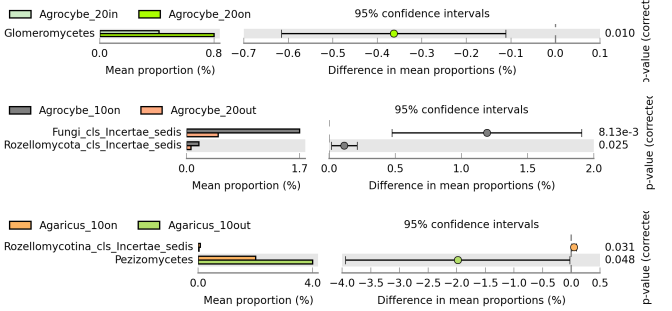


Fig.S10 Analysis of differences at the class level (fungi) in different zones of sub-surface soil in different fairy rings based on the Kruskal-Wallis test (using the Benjamini-Hochberg false discovery rate (FDR) correction).


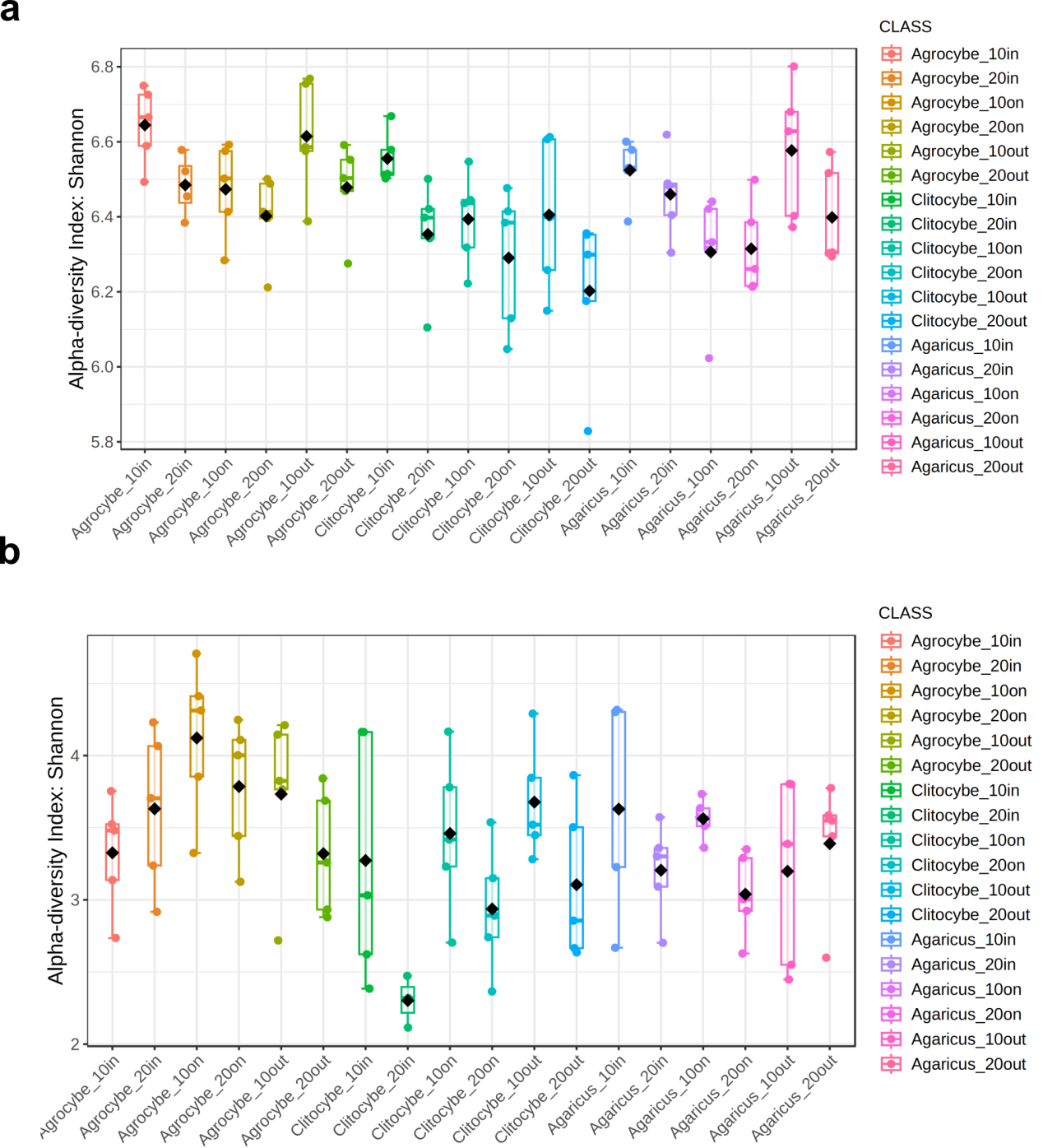
Fig. S11 The Shannon index of bacteria (a) and fungal (b) communities for different fairy rings, zones, and soil depths.


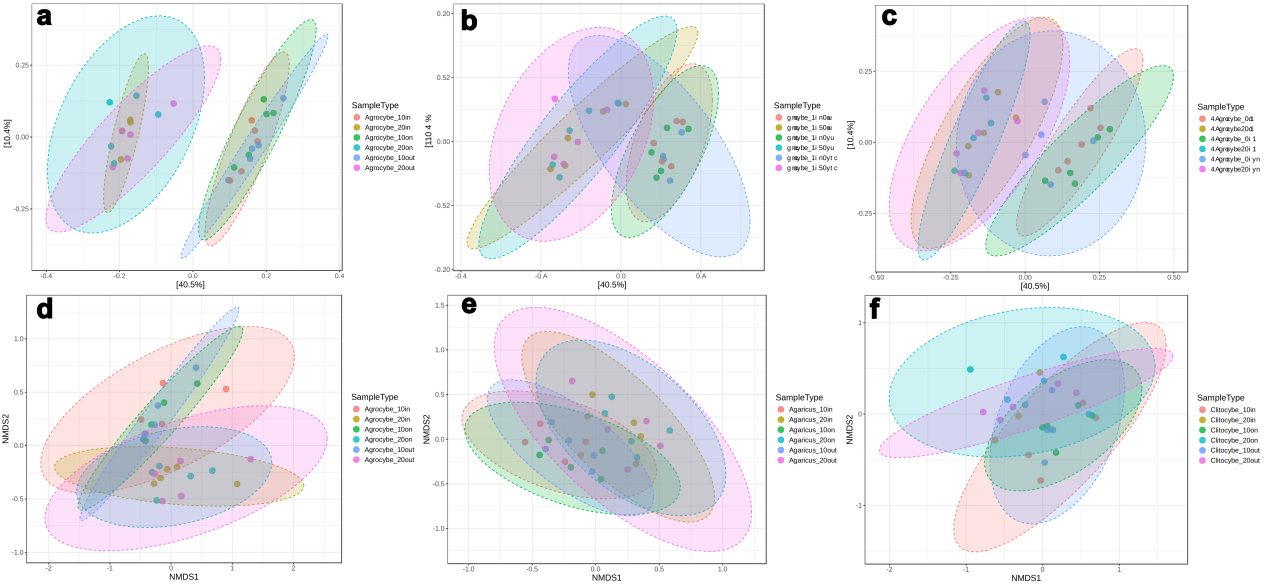


Fig. S12 The non-metric multidimensional scaling (NMDS) based on Bray–Curtis distance matrices for bacteria (a) and fungal (b) communities (a, b, and c are type I, II, and III for bacterial communities, d, e, and f are type I, II, and III for fungal communities).

.


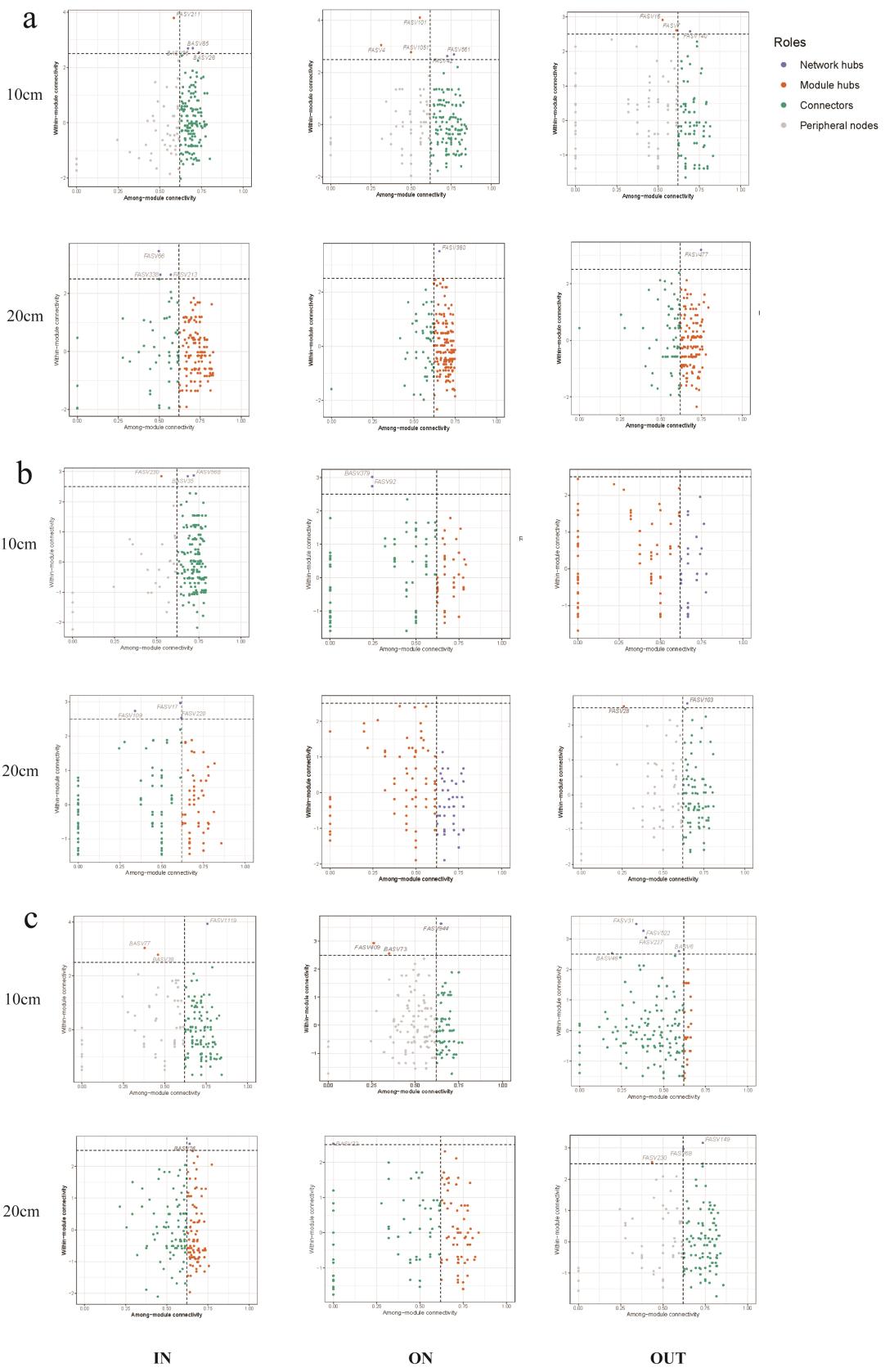


Fig. S13 The co-occurrence network and module hubs for different fairy rings, zones, and soil depths (a, T1; b, T2; c, T3)

Table S1 Sample collection information for three types of fairy ring

| Fairy ring type | Latitude | longitude | altitude | Diameter | date |
| --- | --- | --- | --- | --- | --- |
| T1_1 | 38°32′24.29″ | 99°31′23.19″ | 3537m | 1.27m | 7/20/2020 |
| T1_2 | 38°32′29.16″ | 99°29′44.19″ | 3541m | 1.67m | 7/20/2020 |
| T1_3 | 38°32′26.16″ | 99°29′24.30″ | 3536m | 1.77m | 7/20/2020 |
| T1_4 | 38°32′23.17″ | 99°29′15.19″ | 3537m | 1.92m | 7/21/2020 |
| T1_5 | 38°32′23.31″ | 99°29′20.19″ | 3521m | 2.01. | 7/21/2020 |
| T2_1 | 38°31′19.31″ | 99°28′20.44″ | 3502m | 3.24m | 8/13/2020 |
| T2_2 | 38°31′23.56″ | 99°27′18.20″ | 3421m | 2.97m | 8/13/2020 |
| T2_3 | 38°31′41.68″ | 99°28′20.44″ | 3431m | 3.21m | 8/13/2020 |
| T2_4 | 38°31′18.91″ | 99°28′15.87″ | 3421m | 3.32m | 8/13/2020 |
| T2_5 | 38°31′28.61″ | 99°29′32.44″ | 3423m | 3.49m | 8/13/2020 |
| T3_1 | 38°31′43.95″ | 99°28′19.85″ | 3411m | 2.12m | 9/3/2020 |
| T3_2 | 38°23′09.46″ | 99°27′49.83″ | 3422m | 1.64m | 9/3/2020 |
| T3_3 | 38°25′17.43″ | 99°28′24.23″ | 3433m | 1.72m | 9/3/2020 |
| T3_4 | 38°33′42.36″ | 99°27′02.43″ | 3431m | 1.49m | 9/3/2020 |
| T3_5 | 38°31′66.33″ | 99°28′77.31″ | 3432m | 1.51m | 9/4/2020 |

Table S2 Topology properties of the bacterial intra-kingdom networks.

| Networks | No. Node | No. positive edges | No. negative edges | Average path length | Network diameter | Clustering coefficient | Density | Heterogeneity | Centralization | Modularity |
| --- | --- | --- | --- | --- | --- | --- | --- | --- | --- | --- |
| T1_10_IN | 353 | 783 | 724 | 2.32 | 4.00 | 0.03 | 0.02 | 0.45 | 0.05 | 0.32 |
| T1_10_ON | 353 | 1005 | 825 | 2.08 | 4.00 | 0.03 | 0.03 | 0.43 | 0.06 | 0.28 |
| T1_10_OUT | 342 | 1343 | 1031 | 1.97 | 3.00 | 0.04 | 0.04 | 0.37 | 0.05 | 0.23 |
| T1_20_IN | 377 | 577 | 627 | 2.85 | 6.00 | 0.03 | 0.02 | 0.55 | 0.04 | 0.38 |
| T1_20_ON | 354 | 503 | 487 | 2.73 | 7.00 | 0.02 | 0.02 | 0.50 | 0.03 | 0.42 |
| T1_20_OUT | 371 | 1573 | 1204 | 1.97 | 4.00 | 0.05 | 0.04 | 0.43 | 0.06 | 0.22 |
| T2_10_IN | 353 | 1735 | 1372 | 1.78 | 3.00 | 0.06 | 0.05 | 0.48 | 0.10 | 0.20 |
| T2_10_ON | 375 | 832 | 552 | 2.51 | 5.00 | 0.02 | 0.02 | 0.47 | 0.04 | 0.35 |
| T2_10_OUT | 346 | 2664 | 1547 | 1.64 | 3.00 | 0.09 | 0.07 | 0.40 | 0.10 | 0.18 |
| T2_20_IN | 359 | 1009 | 1127 | 2.03 | 4.00 | 0.04 | 0.03 | 0.44 | 0.06 | 0.26 |
| T2_20_ON | 361 | 3522 | 1632 | 1.54 | 3.00 | 0.10 | 0.08 | 0.43 | 0.14 | 0.16 |
| T2_20_OUT | 343 | 725 | 1047 | 2.12 | 4.00 | 0.04 | 0.03 | 0.39 | 0.04 | 0.28 |
| T3_10_IN | 353 | 734 | 878 | 2.23 | 5.00 | 0.03 | 0.03 | 0.46 | 0.04 | 0.31 |
| T3_10_ON | 378 | 422 | 757 | 2.59 | 5.00 | 0.02 | 0.02 | 0.47 | 0.03 | 0.39 |
| T3_10_OUT | 360 | 1406 | 819 | 1.99 | 4.00 | 0.04 | 0.03 | 0.42 | 0.05 | 0.26 |
| T3_20_IN | 366 | 1124 | 1730 | 1.87 | 4.00 | 0.06 | 0.04 | 0.48 | 0.09 | 0.22 |
| T3_20_ON | 369 | 1131 | 1003 | 2.09 | 4.00 | 0.04 | 0.03 | 0.42 | 0.04 | 0.27 |
| T3_20_OUT | 362 | 626 | 457 | 2.80 | 6.00 | 0.02 | 0.02 | 0.58 | 0.05 | 0.40 |

Table S3 Topology properties of the fungal intra-kingdom networks.

| Networks | No. Node | No. positive edges | No. negative edges | Average path length | Network diameter | Clustering coefficient | Density | Heterogeneity | Centralization | Modularity |
| --- | --- | --- | --- | --- | --- | --- | --- | --- | --- | --- |
| T1_10_IN | 154 | 190 | 176 | 2.76 | 6.00 | 0.03 | 0.03 | 0.51 | 0.06 | 0.43 |
| T1_10_ON | 196 | 314 | 499 | 2.40 | 5.00 | 0.05 | 0.04 | 0.47 | 0.06 | 0.34 |
| T1_10_OUT | 180 | 237 | 308 | 2.30 | 4.00 | 0.05 | 0.04 | 0.44 | 0.08 | 0.31 |
| T1_20_IN | 158 | 231 | 307 | 2.25 | 4.00 | 0.04 | 0.04 | 0.42 | 0.07 | 0.32 |
| T1_20_ON | 181 | 334 | 371 | 2.62 | 5.00 | 0.03 | 0.03 | 0.50 | 0.07 | 0.36 |
| T1_20_OUT | 135 | 140 | 228 | 2.61 | 5.00 | 0.04 | 0.04 | 0.41 | 0.06 | 0.40 |
| T2_10_IN | 169 | 313 | 451 | 2.06 | 4.00 | 0.06 | 0.05 | 0.41 | 0.06 | 0.30 |
| T2_10_ON | 133 | 95 | 192 | 1.80 | 3.00 | 0.10 | 0.10 | 0.32 | 0.08 | 0.23 |
| T2_10_OUT | 115 | 63 | 102 | 2.17 | 4.00 | 0.06 | 0.06 | 0.42 | 0.07 | 0.28 |
| T2_20_IN | 133 | 95 | 192 | 1.80 | 3.00 | 0.10 | 0.10 | 0.32 | 0.08 | 0.23 |
| T2_20_ON | 154 | 284 | 398 | 3.00 | 5.00 | 0.04 | 0.03 | 0.48 | 0.05 | 0.46 |
| T2_20_OUT | 127 | 42 | 128 | 4.01 | 9.00 | 0.00 | 0.02 | 0.64 | 0.05 | 0.63 |
| T3_10_IN | 155 | 385 | 504 | 1.86 | 3.00 | 0.08 | 0.07 | 0.39 | 0.09 | 0.25 |
| T3_10_ON | 156 | 273 | 283 | 2.90 | 6.00 | 0.03 | 0.03 | 0.49 | 0.04 | 0.45 |
| T3_10_OUT | 144 | 156 | 240 | 2.28 | 4.00 | 0.06 | 0.05 | 0.45 | 0.06 | 0.34 |
| T3_20_IN | 169 | 1 | 1 | 7.45 | 20.00 | 0.02 | 0.02 | 0.55 | 0.04 | 0.79 |
| T3_20_ON | 92 | 27 | 63 | 2.54 | 6.00 | 0.05 | 0.04 | 0.52 | 0.07 | 0.42 |
| T3_20_OUT | 104 | 81 | 81 | 3.50 | 8.00 | 0.03 | 0.03 | 0.60 | 0.08 | 0.58 |

Table S4 Topology properties of the inter-kingdom networks.

|  |  | interaction_type | Positive | Negative | total | positive_ratio | negative_ratio |
| --- | --- | --- | --- | --- | --- | --- | --- |
| T1 | 10in | bacteria-bacteria | 541 | 188 | 729 | 74.21% | 25.79% |
|  |  | bacteria-fungi | 550 | 511 | 1061 | 51.93% | 48.16% |
|  |  | fungi-fungi | 254 | 167 | 421 | 60.33% | 39.67% |
|  | 10on | bacteria-bacteria | 14 | 17 | 31 | 45.16% | 54.84% |
|  |  | bacteria-fungi | 116 | 145 | 261 | 44.44% | 55.56% |
|  |  | fungi-fungi | 247 | 364 | 611 | 40.43% | 59.57% |
|  | 10out | bacteria-bacteria | 2 | 26 | 28 | 7.14% | 92.86% |
|  |  | bacteria-fungi | 69 | 132 | 201 | 34.33% | 65.67% |
|  |  | fungi-fungi | 115 | 150 | 265 | 44.40% | 58.59% |
|  | 20in | bacteria-bacteria | 36 | 52 | 88 | 40.91% | 59.09% |
|  |  | bacteria-fungi | 150 | 293 | 433 | 34.64% | 47.67% |
|  |  | fungi-fungi | 161 | 233 | 394 | 40.86% | 59.14% |
|  | 20on | bacteria-bacteria | 120 | 165 | 285 | 42.11% | 57.89% |
|  |  | bacteria-fungi | 436 | 396 | 832 | 52.40% | 47.60% |
|  |  | fungi-fungi | 421 | 413 | 834 | 50.48% | 49.52% |
|  | 20out | bacteria-bacteria | 368 | 221 | 589 | 62.48% | 37.52% |
|  |  | bacteria-fungi | 299 | 460 | 759 | 39.39% | 60.61% |
|  |  | fungi-fungi | 166 | 174 | 340 | 48.82% | 51.12% |
| T2 | 10in | bacteria-bacteria | 152 | 64 | 216 | 70.37% | 29.63% |
|  |  | bacteria-fungi | 369 | 368 | 737 | 50.07% | 49.93% |
|  |  | fungi-fungi | 341 | 370 | 711 | 47.96% | 52.04% |
|  | 10on | bacteria-bacteria | 3 | 0 | 3 | 100% | 0% |
|  |  | bacteria-fungi | 23 | 38 | 61 | 37.70% | 62.30% |
|  |  | fungi-fungi | 80 | 171 | 251 | 30.87% | 68.13% |
|  | 10out | bacteria-bacteria | 2 | 19 | 21 | 9.52% | 90.48% |
|  |  | bacteria-fungi | 32 | 80 | 112 | 28.57% | 71.43% |
|  |  | fungi-fungi | 37 | 78 | 115 | 32.17% | 67.83.% |
|  | 20in | bacteria-bacteria | 12 | 14 | 26 | 46.15% | 53.85% |
|  |  | bacteria-fungi | 40 | 76 | 116 | 34.48% | 65.52% |
|  |  | fungi-fungi | 73 | 153 | 226 | 32.30% | 67.70% |
|  | 20on | bacteria-bacteria | 7 | 6 | 13 | 53.85% | 46.15% |
|  |  | bacteria-fungi | 100 | 79 | 179 | 55.87% | 44.13% |
|  |  | fungi-fungi | 91 | 117 | 208 | 43.75% | 56.25% |
|  | 20out | bacteria-bacteria | 16 | 55 | 71 | 22.54% | 77.46% |
|  |  | bacteria-fungi | 109 | 203 | 312 | 34.94% | 65.06% |
|  |  | fungi-fungi | 117 | 196 | 296 | 39.53% | 60.47% |
| T3 | 10in | bacteria-bacteria | 93 | 77 | 170 | 54.71% | 45.29% |
|  |  | bacteria-fungi | 188 | 224 | 412 | 45.63% | 54.37% |
|  |  | fungi-fungi | 67 | 94 | 161 | 41.61% | 58.39% |
|  | 10on | bacteria-bacteria | 196 | 53 | 249 | 78.71% | 21.29% |
|  |  | bacteria-fungi | 353 | 217 | 570 | 61.93% | 38.07% |
|  |  | fungi-fungi | 241 | 251 | 492 | 48.98% | 51.02% |
|  | 10out | bacteria-bacteria | 269 | 26 | 295 | 91.19% | 8.81% |
|  |  | bacteria-fungi | 465 | 125 | 590 | 78.81% | 21.19% |
|  |  | fungi-fungi | 208 | 123 | 331 | 62.84% | 37.16% |
|  | 20in | bacteria-bacteria | 570 | 151 | 721 | 79.06% | 20.94% |
|  |  | bacteria-fungi | 747 | 210 | 957 | 78.06% | 21.94% |
|  |  | fungi-fungi | 121 | 61 | 182 | 66.48% | 33.52% |
|  | 20on | bacteria-bacteria | 45 | 34 | 79 | 56.96% | 43.04% |
|  |  | bacteria-fungi | 103 | 98 | 201 | 51.24% | 48.76% |
|  |  | fungi-fungi | 81 | 104 | 185 | 43.78% | 56.22% |
|  | 20out | bacteria-bacteria | 113 | 44 | 157 | 71.97% | 28.03% |
|  |  | bacteria-fungi | 181 | 151 | 332 | 54.52% | 45.48% |
|  |  | fungi-fungi | 85 | 104 | 189 | 44.97% | 55.03% |
