# Supplementary material for: Microbial interactions mediate the fairy ring type effects on alpine meadow plant communities on the Tibetan plateau
Source: Environ Microbiome. 2026 Mar 10;21:55. doi: 10.1186/s40793-026-00873-z (PMC13088534; doi:10.1186/s40793-026-00873-z)
Supplement: Supplementary file 2 — Supplementary Material 2 [file 40793_2026_873_MOESM2_ESM.zip › lefse/bacteria/T1.pdf]

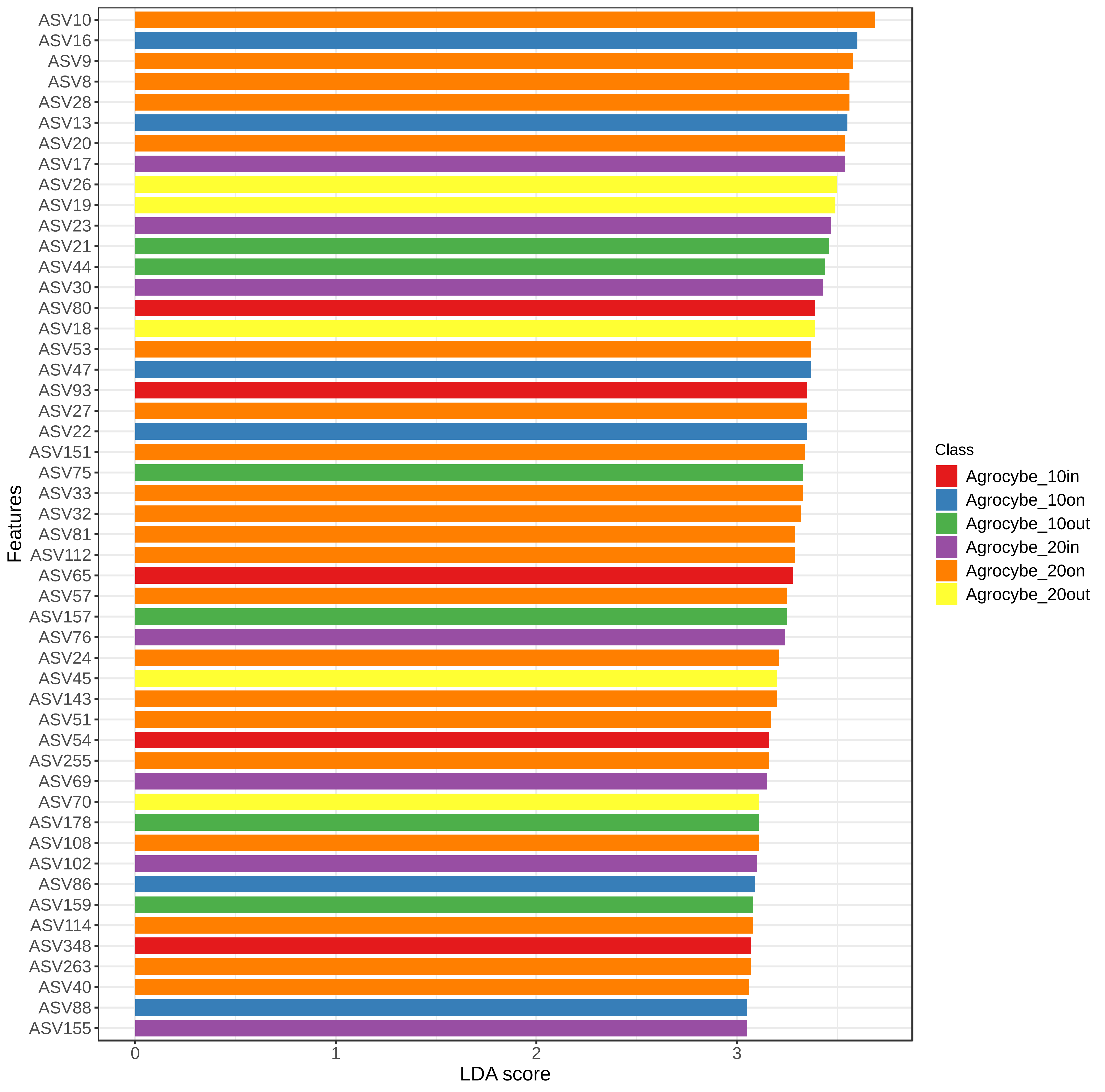

ASV10
ASV16
ASV9
ASV8
ASV28
ASV13
ASV20
ASV17
ASV26
ASV19
ASV23
ASV21
ASV44
ASV30
ASV80
ASV18
ASV53
ASV47
ASV93
ASV27
ASV22
ASV151
ASV75
ASV33
ASV32
ASV81
ASV112
ASV65
ASV57
ASV157
ASV76
ASV24
ASV45
ASV143
ASV51
ASV54
ASV255
ASV69
ASV70
ASV178
ASV108
ASV102
ASV86
ASV159
ASV114
ASV348
ASV263
ASV40
ASV88
ASV155
Features
0
1
2
3
LDA score
Class
Agrocybe_10in
Agrocybe_10on
Agrocybe_10out
Agrocybe_20in
Agrocybe_20on
Agrocybe_20out
