# Supplementary material for: Microbial interactions mediate the fairy ring type effects on alpine meadow plant communities on the Tibetan plateau
Source: Environ Microbiome. 2026 Mar 10;21:55. doi: 10.1186/s40793-026-00873-z (PMC13088534; doi:10.1186/s40793-026-00873-z)
Supplement: Supplementary file 2 — Supplementary Material 2 [file 40793_2026_873_MOESM2_ESM.zip › lefse/bacteria/T2.pdf]

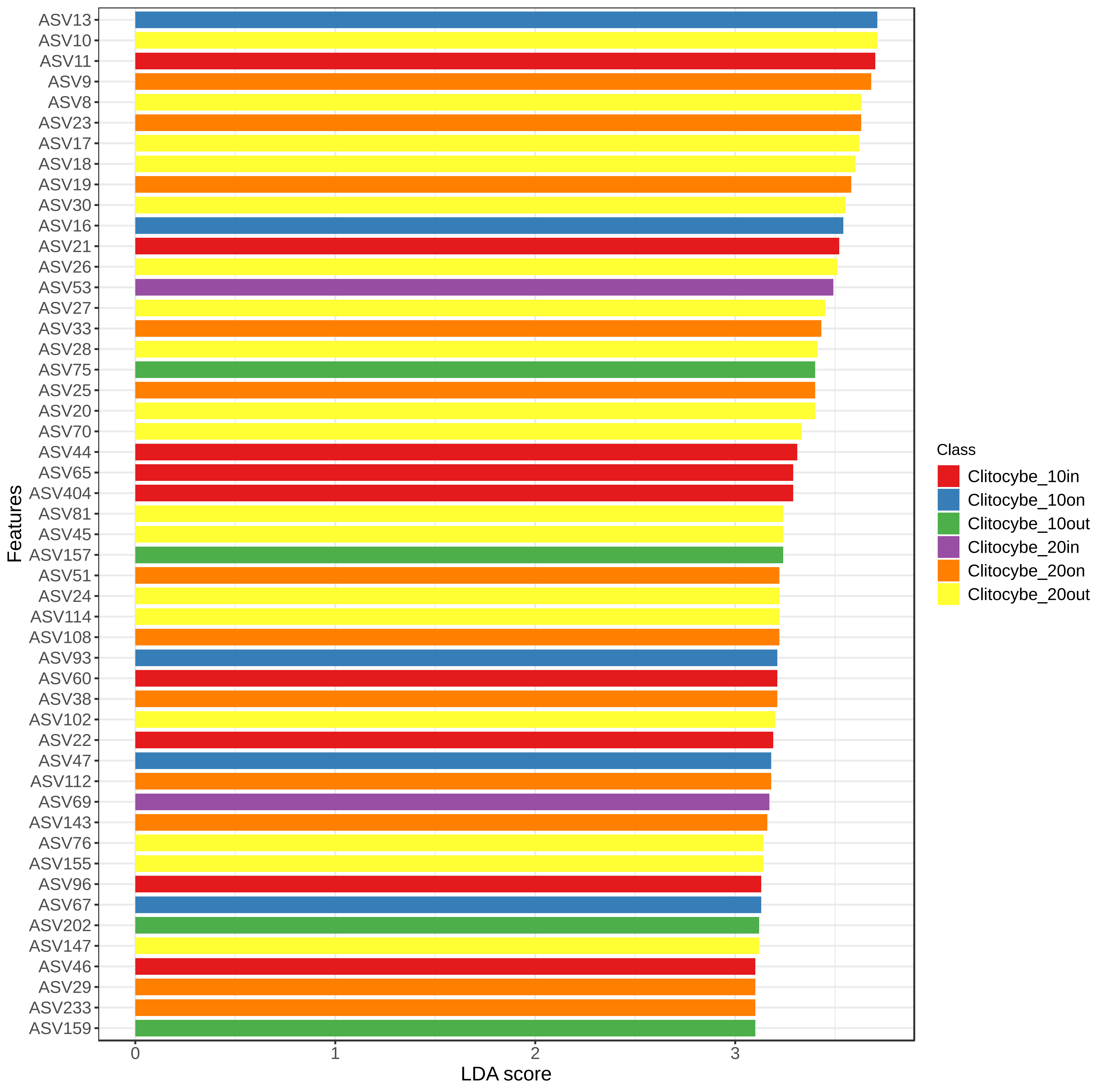

ASV13
ASV10
ASV11
ASV9
ASV8
ASV23
ASV17
ASV18
ASV19
ASV30
ASV16
ASV21
ASV26
ASV53
ASV27
ASV33
ASV28
ASV75
ASV25
ASV20
ASV70
ASV44
ASV65
ASV404
ASV81
ASV45
ASV157
ASV51
ASV24
ASV114
ASV108
ASV93
ASV60
ASV38
ASV102
ASV22
ASV47
ASV112
ASV69
ASV143
ASV76
ASV155
ASV96
ASV67
ASV202
ASV147
ASV46
ASV29
ASV233
ASV159
Features
0
1
2
3
LDA score
Class
Clitocybe_10in
Clitocybe_10on
Clitocybe_10out
Clitocybe_20in
Clitocybe_20on
Clitocybe_20out
