# Supplementary material for: Microbial interactions mediate the fairy ring type effects on alpine meadow plant communities on the Tibetan plateau
Source: Environ Microbiome. 2026 Mar 10;21:55. doi: 10.1186/s40793-026-00873-z (PMC13088534; doi:10.1186/s40793-026-00873-z)
Supplement: Supplementary file 2 — Supplementary Material 2 [file 40793_2026_873_MOESM2_ESM.zip › lefse/bacteria/T3.pdf]

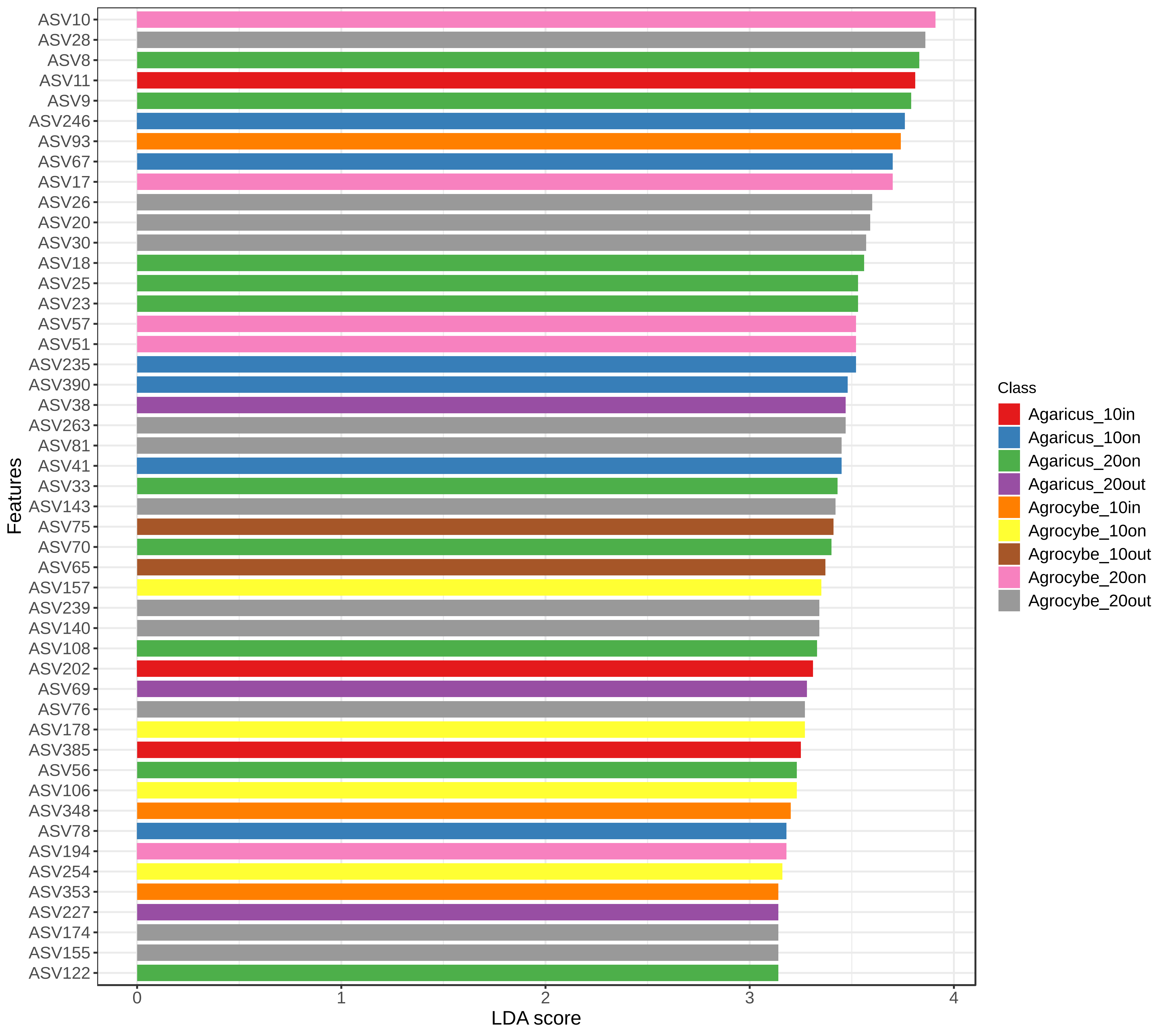

ASV10
ASV28
ASV8
ASV11
ASV9
ASV246
ASV93
ASV67
ASV17
ASV26
ASV20
ASV30
ASV18
ASV25
ASV23
ASV57
ASV51
ASV235
ASV390
ASV38
ASV263
ASV81
ASV41
ASV33
ASV143
ASV75
ASV70
ASV65
ASV157
ASV239
ASV140
ASV108
ASV202
ASV69
ASV76
ASV178
ASV385
ASV56
ASV106
ASV348
ASV78
ASV194
ASV254
ASV353
ASV227
ASV174
ASV155
ASV122
Features
0
1
2
3
4
LDA score
Class
Agaricus_10in
Agaricus_10on
Agaricus_20on
Agaricus_20out
Agrocybe_10in
Agrocybe_10on
Agrocybe_10out
Agrocybe_20on
Agrocybe_20out
