# Supplementary material for: Microbial interactions mediate the fairy ring type effects on alpine meadow plant communities on the Tibetan plateau
Source: Environ Microbiome. 2026 Mar 10;21:55. doi: 10.1186/s40793-026-00873-z (PMC13088534; doi:10.1186/s40793-026-00873-z)
Supplement: Supplementary file 2 — Supplementary Material 2 [file 40793_2026_873_MOESM2_ESM.zip › lefse/fungi/T1_bar_graph_5.pdf]

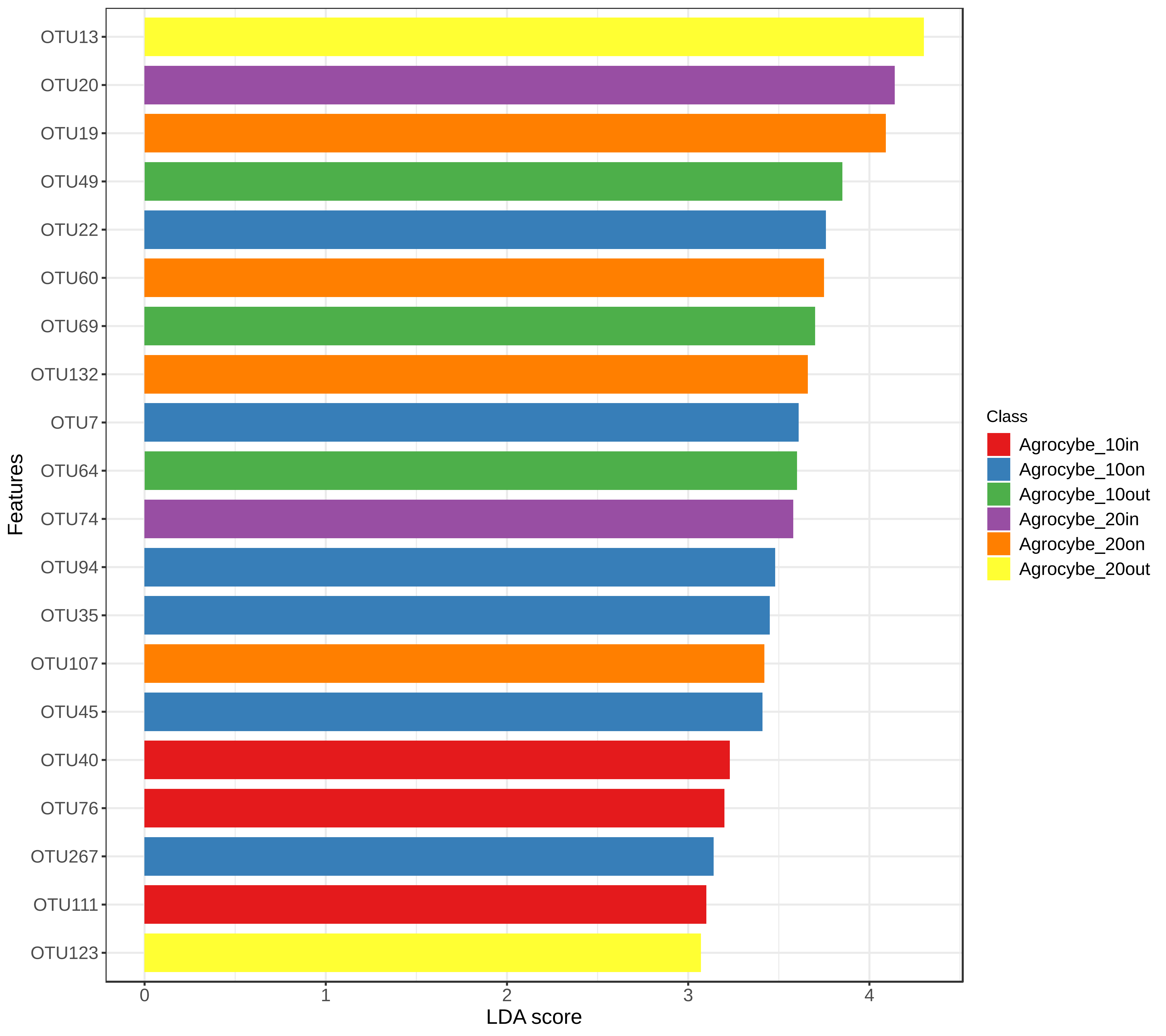
OTU13
OTU20
OTU19
OTU49
OTU22
OTU60
OTU69
OTU132
OTU7
OTU64
OTU74
OTU94
OTU35
OTU107
OTU45
OTU40
OTU76
OTU267
OTU111
OTU123
Features
0
1
2
3
4
LDA score
Class
Agrocybe_10in
Agrocybe_10on
Agrocybe_10out
Agrocybe_20in
Agrocybe_20on
Agrocybe_20out
