# Supplementary material for: Microbial interactions mediate the fairy ring type effects on alpine meadow plant communities on the Tibetan plateau
Source: Environ Microbiome. 2026 Mar 10;21:55. doi: 10.1186/s40793-026-00873-z (PMC13088534; doi:10.1186/s40793-026-00873-z)
Supplement: Supplementary file 2 — Supplementary Material 2 [file 40793_2026_873_MOESM2_ESM.zip › lefse/fungi/T2_bar_graph_5.pdf]

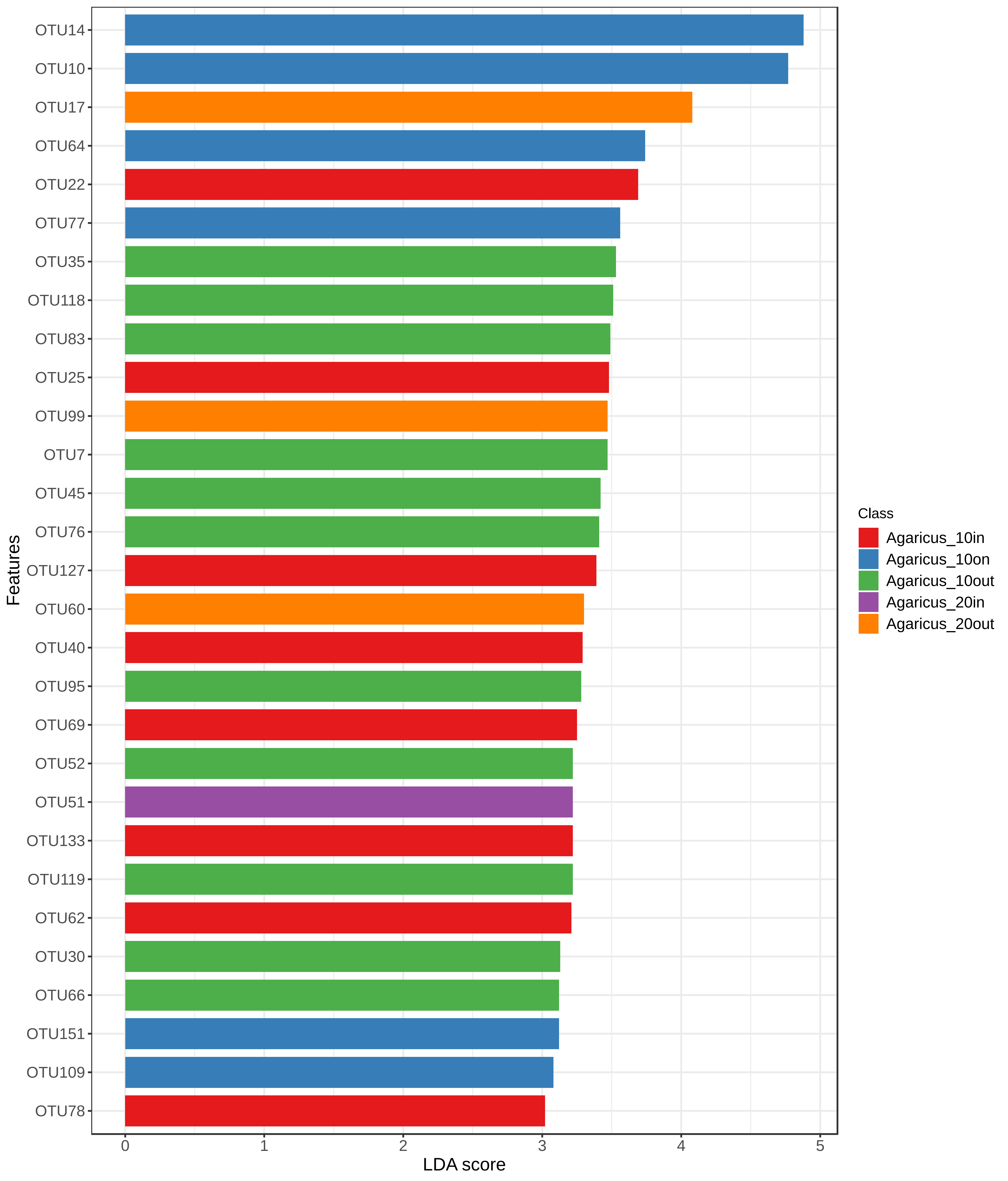
OTU14
OTU10
OTU17
OTU64
OTU22
OTU77
OTU35
OTU118
OTU83
OTU25
OTU99
OTU7
OTU45
OTU76
OTU127
OTU60
OTU40
OTU95
OTU69
OTU52
OTU51
OTU133
OTU119
OTU62
OTU30
OTU66
OTU151
OTU109
OTU78
Features
0
1
2
3
4
5
LDA score
Class
Agaricus_10in
Agaricus_10on
Agaricus_10out
Agaricus_20in
Agaricus_20out
